# Benefits of Digital Health Resources for Substance Use Concerns in Women: Scoping Review

**DOI:** 10.2196/25952

**Published:** 2021-06-07

**Authors:** Lena Quilty, Branka Agic, Michelle Coombs, Betty-Lou Kristy, Jill Shakespeare, Adrienne Spafford, Reena Besa, Shadini Dematagoda, Alina Patel, Rebecca Persaud, Leslie Buckley

**Affiliations:** 1 Centre for Addiction and Mental Health Toronto, ON Canada; 2 University of Toronto Toronto, ON Canada; 3 The Jean Tweed Treatment Centre Toronto, ON Canada; 4 Centre for Innovation in Peer Support Support House Oakville, ON Canada; 5 Addictions and Mental Health Ontario Toronto, ON Canada

**Keywords:** women, female, gender-specific, digital health, internet, mobile app, technology, technology interventions, technology-based intervention, web-based intervention, substance use concerns, trauma

## Abstract

**Background:**

Digital health resources are being increasingly used to support women with substance use concerns. Although empirical research has demonstrated that these resources have promise, the available evidence for their benefit in women requires further investigation. Evidence supports the capacity of interventions that are sex-, gender-, and trauma-informed to improve treatment access and outcomes and to reduce health system challenges and disparities. Indeed, both sex- and gender-specific approaches are critical to improve health and gender equity. Violence and trauma are frequent among those with substance use concerns, but they disproportionately affect those who identify as female or women, further underscoring the need for trauma-informed care as well.

**Objective:**

The objective of this investigation was to evaluate the evidence supporting the efficacy or effectiveness of online or mobile interventions for risky or harmful substance use in adults who identify as female or women, or who report a history of trauma.

**Methods:**

This scoping review is based on an academic search in MEDLINE, APA PsycINFO, Embase, Cochrane Central, and CINAHL, as well as a grey literature search in US and Canadian government and funding agency websites. Of the 7807 records identified, 465 remained following title and abstract screening. Of these, 159 met all eligibility criteria and were reviewed and synthesized.

**Results:**

The 159 records reflected 141 distinct studies and 125 distinct interventions. Investigations and the interventions evaluated predominantly focused on alcohol use or general substance use. Evaluated digital health resources included multisession and brief-session interventions, with a wide range of therapeutic elements. Multisession online and mobile interventions exhibited beneficial effects in 86.1% (105/122) of studies. Single-session interventions similarly demonstrated beneficial effects in 64.2% (43/67) of study conditions. Most investigations did not assess gender identity or conduct sex- or gender-based analyses. Only 13 investigations that included trauma were identified.

**Conclusions:**

Despite the overall promise of digital health interventions for substance use concerns, direct or quantitative evidence on the efficacy or effectiveness of interventions in females or women specifically is weak.

## Introduction

### Background

Despite the higher prevalence of substance misuse and substance use disorders in men compared with women, a substantial proportion of women do experience harms associated with substance use. Moreover, research suggests that substance use and associated harms have been increasing in women over time. For example, the frequency and volume of alcohol use in women increased substantially from the 2000s to 2010s [1,2]. Cannabis use has exhibited an increase over even shorter time periods, and recent estimates suggest that 10% of women in Canada self-reported having a dependence on some form of illicit drug [1,3]. Substance use in women is further associated with staggering personal and societal costs. In particular, it is strongly linked to mental health concerns, including depression and suicidal thoughts and behaviors [4], as well as physical health concerns, including morbidity and mortality [5]. Substance misuse has associated health impacts on maternal health, fetal and neonatal morbidity, prematurity, and small for gestational age. It also leads to parenting deficits related to psychological and environmental concerns [6,7]. Overall, societal costs associated with substance use are widespread and growing, as illustrated by increasing hospitalizations due to substance use [8] and increasing loss in productivity, which is estimated to be over Can $15 billion [7].

### Sex-, Gender-, and Trauma-Informed Supports for Substance Use Concerns

Despite an increase in substance use among women, women are generally underrepresented in treatment settings [9]. Research has suggested that women are less inclined to seek treatment until negative consequences become severe [10,11]. Additionally, research has demonstrated that women experience specific barriers to care, from psychological barriers, such as stigma and discrimination, to practical barriers, such as decreased opportunity due to caregiving roles and responsibilities, relationship abuse and violence, etc [12]. Women are more likely to be principal caregivers to children and other family members, and concerns regarding the potential involvement of child protection services or other social services as a result of seeking support can be a particularly powerful deterrent. These barriers can thus delay treatment seeking, such that women presenting to specialized services exhibit both acute and complex needs to impact both treatment engagement and outcomes.

Evidence supports the capacity of interventions that are sex-, gender-, and trauma-informed to improve treatment access and outcomes and to reduce health system challenges and disparities. Indeed, both sex- and gender-specific approaches are critical to improve health and gender equity, attending to the biological factors that impact the response to substances and biological treatments, as well as the gendered experiences of substance use challenges and their management [13]. Trauma is critical to consider in this context. While specifics may vary, trauma is generally defined as an emotional consequence of a deeply distressing or disturbing event [14] that has overcome an individual’s ability to cope [13]. An elevated prevalence of substance use among those with a history of trauma supports a strong overall association between trauma exposure and substance misuse [15]. Strikingly, 75% of women and more than 25% of men who enter treatment for substance use disorders report histories of abuse and trauma [15-17]. Those with a history of trauma have been shown to experience more complications in treatment for substance use disorders, with higher levels of distress, lower treatment adherence, and longer courses, when compared with those without a history of trauma [18]. Despite high prevalence rates and significant implications, trauma is not frequently assessed or addressed in the treatment of substance use disorders [19]. Thus, although trauma and substance use concerns frequently co-occur, adults who identify as female or women are disproportionately affected by trauma and the impact of trauma on care. This health disparity further underscores the need for sex-, gender-, and trauma-informed interventions.

Current evidence-based best practice guidelines have therefore highlighted the importance of gender- and trauma-informed treatments for substance use concerns in women. Gender-informed practices include integrated treatment approaches addressing a wide range of women’s needs (eg, physical, social, and mental health needs, and child-centered services such as prenatal services, parenting programs, and child care) and are associated with improvements in recovery, parenting skills, and emotional health [20]. Trauma-informed practices, in turn, follow the principles of trauma awareness/acknowledgment; maintaining trust and safety; promoting choice and collaboration; maintaining focus on strength/skills building; attending to cultural, historical, and gender issues such as intimate partner violence; peer support; and mutual self-help. Trauma-informed care is also associated with improved service user experiences and clinical outcomes [21].

It is notable then that the gender- and trauma-informed practices most appropriate to women with substance use difficulties primarily comprise integrated psychosocial interventions, most commonly provided in-person and in group formats. Yet, in many jurisdictions, this model of care delivery is not possible to maintain during the COVID-19 global pandemic. Similar to other health care settings, substance use treatment centers serving women are increasingly turning to digital health solutions to provide support, particularly while physical distancing measures are necessary to protect public health. Digital health solutions may in fact overcome numerous barriers to care experienced by women and provide a valuable addition to the health system even beyond the current crisis.

In a recent review, Nesvåg and McKay [22] evaluated the feasibility and therapeutic benefits of digital interventions to prevent and treat substance use concerns. This review located 28 unique interventions, which were categorized as simple or complex based on the number of features. Simple interventions were generally mobile apps integrated within other services and supports, whereas complex interventions were more frequently delivered as stand-alone interventions, using a personal computer and/or a mobile app format. A large proportion of participants (70%-90%) found the interventions to be useful, and more than half of the studies found small to medium positive effects in comparison to a control group. This review supported the feasibility of digital health resources for substance use concerns, but found less consistent support for their efficacy or effectiveness. In a review centered on women of childbearing age, Hai et al [23] evaluated the efficacy of technology-based interventions for substance use, with a focus on randomized controlled trials. This review located 15 trials, and a meta-analysis of 13 trials supported the efficacy of the digital health interventions for alcohol use concerns specifically compared with control conditions.

This review extends the foundational work in several ways. First, Hai et al [23] specifically focused on studies conducted in women of childbearing age, precluding an evaluation of differential effects across sex or gender. Second, both Hai et al [23] and Nesvåg and McKay [22] specifically focused on randomized controlled trials; however, initial investigations as well as investigations with a focus on effectiveness and/or implementation outcomes in real-world settings may utilize different research designs. Third, previous reviews have not systematically extracted data regarding the trauma endorsed by samples, limiting the capacity to determine the degree to which this crucial clinical feature is integrated into research designs, analyses, and interpretations. The current investigation therefore conducted a scoping review to evaluate the nature of the evidence for the efficacy and/or effectiveness of digital health resources to treat substance use and/or associated risks or harms. Consistent with recommendations [24], we conducted a scoping review to evaluate the types of available evidences in the field, which we envisioned would therefore either act as a precursor to a systematic review or support the analysis of knowledge gaps, contingent upon the results. We therefore implemented a search strategy including a wide range of research designs and requiring a limited proportion of adults who identify as female or women, or who report a history of trauma, regardless of sex or gender. We focused on web-based interventions as classified by Barak et al [25], specifically self-guided interventions with or without adjunctive tailored human support. We did not incorporate remotely delivered synchronous interventions due to stakeholder-identified needs for digital interventions that do not necessitate clinician mediation or delivery and that may extend the capacity of the limited workforce to meet increasing clinical demands [26].

The aim of this investigation was to evaluate the current evidence for digital health resources for substance use concerns, with a focus on resources that have been evaluated in females or women, or in those who report a history of trauma, regardless of sex or gender. Although current resources may not have been designed to fully incorporate gender- and trauma-based principles, their therapeutic benefit in these groups is nevertheless an important consideration in evaluating currently available resources, as well as identifying priorities for both clinical and research initiatives.

## Methods

### Overview

The methodology for this scoping review was based on the framework developed by Arksey and O’Malley [27] and later refined by Levac et al [28]. The stages are briefly outlined as (1) identifying the research question, (2) identifying relevant studies, (3) study selection, (4) charting the data, and (5) collating, summarizing, and reporting the resources. Each stage is described below.

### Stage 1: Identifying the Research Question

The scoping review was conducted to answer the following research questions:

1. What digital health resources have been evaluated in those who identify as female/women or those reporting a history of trauma, regardless of sex or gender?
2. What digital health resources have empirical support for their efficacy/effectiveness in those who identify as female/women or those reporting a history of trauma, regardless of sex or gender?

For the purpose of this study, a scoping review was defined as a type of research synthesis that aims to “map the literature” on a particular topic or research area and provide an opportunity to identify key concepts; gaps in the research; and types and sources of evidence to inform practice, policymaking, and research [29]. Through answering the above questions our objective was to evaluate the nature of the evidence base for the efficacy and/or effectiveness of digital health resources for reducing substance use and/or associated harms in those identifying as female/women or in those reporting a history of trauma, regardless of sex or gender.

### Stage 2: Identifying Relevant Studies

A comprehensive search strategy was developed by a librarian (RB) in consultation with the research team. The following databases were searched from inception: MEDLINE (including Epub ahead of print, in-process, and other nonindexed citations), APA PsycINFO, Embase, Cochrane Central, and CINAHL. No language limits were applied at this stage. For the searches, combinations of controlled vocabulary in the form of database-specific subject headings and relevant free-text keywords were included. The database searches were conducted in June 2020. The full MEDLINE search strategy is available for viewing in Multimedia Appendix 1.

In addition, nonpeer reviewed (grey) literature was also retrieved. The research team conducted a web search of Canadian and US Government and Funding Agencies in Canada and the United States using Google from July to August 2020. These searches were conducted using variations of the following (including but not limited to): “substance use,” “drug use,” “alcohol use,” or “addiction;” “online intervention,” “digital health,” “eHealth,” or “mobile health;” and “women” or “female.”

### Stage 3: Study Selection

Studies were selected according to the following eligibility criteria:

1. Language: We included articles in English.
2. Date: We included articles from database inception to the date of extraction (June 30, 2020).
3. Publication type: We only considered original research articles, including secondary analyses. Dissertations, commentaries, conference proceedings, letters, editorials, and reviews were excluded to ensure presence of sufficient methodology and data needed to map and evaluate the nature of the evidence.
4. Sample: We considered adults aged 18 years or older, who endorsed or exhibited risky or harmful substance use. Similar to previous reviews [22,23], we did not include nicotine or caffeine. A minimum of 20% of participants was required to identify as female and/or women, or to report a trauma history, regardless of sex or gender. Although in many contexts, a higher proportion would be more appropriate and/or necessary to ensure power of sex- or gender-based analyses, this lower limit permits broad sampling of evidence to evaluate current practices.
5. Setting: We considered all settings (eg, health care, forensic, and educational).
6. Design: We included all prospective designs (eg, single vs multiple arms and augmentation vs stand-alone intervention). Randomization or a comparison/control group was not required.
7. Intervention: We considered web- or mobile-based interventions targeting substance use or substance use disorder symptoms. All theoretical orientations and durations of treatments were included; however, formats that were computer-based, but not online, or that were interactive were excluded (eg, telephone, video, and text-based interactive psychosocial interventions with a clinician and social networking/platforms such as peer support discussion boards).
8. Outcomes: We considered substance use or substance use disorder symptoms. Outcomes that were focused only on acceptability or feasibility were excluded.

Following the initial extraction and removal of duplicates, two research staff independently (1) screened the titles and abstracts of all unique records, (2) conducted full-text reviews for all records not excluded, and (3) extracted data from included studies. Team members demonstrated substantial agreement during title and abstract screening (96% agreement; κ=0.74) and during the full-text review (92% agreement; κ=0.84). Discrepancies were resolved by consensus, with the support of a member of the investigation team as needed (LQ).

### Stage 4: Charting the Data

Procedures were consistent with Preferred Reporting Items for Systematic Reviews and Meta-Analyses (PRISMA) extension for Scoping Reviews (PRISMA-ScR) guidelines [30]. The following data were extracted from records included in synthesis: study design features (eg, setting and randomized controlled trial), sample features (eg, size and demographic information), intervention (eg, duration and components), outcomes (eg, instruments and indicators), and bias and fidelity indicators. Two research staff independently extracted data, and discrepancies were resolved by consensus.

### Stage 5: Collating, Summarizing, and Reporting the Results

The Cochrane Risk of Bias Tool was used to evaluate bias at the study level across the following six domains: sequence generation, allocation concealment, blinding, incomplete data, selective reporting, and overall risk [31]. Each domain was given a rating of high, low, or some concerns of bias.

## Results

### Study Identification

**Figure 1:**
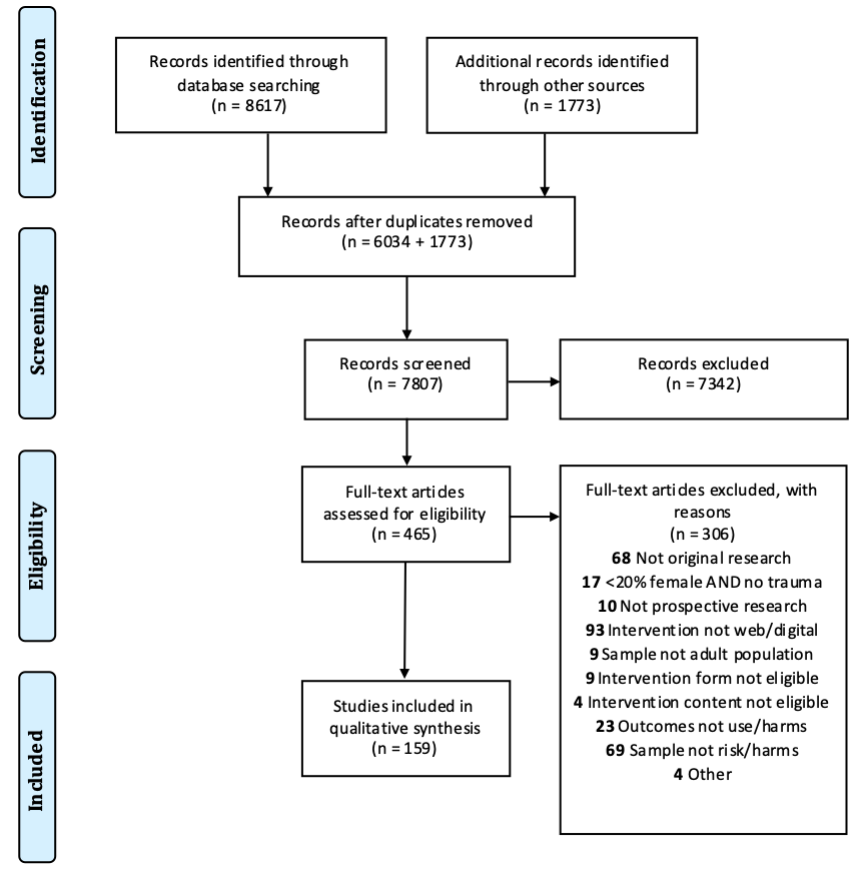
PRISMA (Preferred Reporting Items for Systematic Reviews and Meta-Analyses) flow diagram.

The study selection process is illustrated in Figure 1. We located a total of 8617 published and 1773 grey literature records. A total of 7807 records remained after removing duplicates, and then, 465 remained following the screening of titles and abstracts. Records excluded at this first stage most commonly did not report original data or did not include critical design (eg, not original research and not a prospective design), sample (eg, adults with harmful/risk substance use), or intervention features (eg, online/mobile intervention targeting substance use or harms). Of the 465 remaining records, 306 were excluded because they did not include original (n=68) or prospective (n=10) research; did not include an adult sample (n=9) endorsing substance use risk or harms (n=69) with the minimum proportion of females/women or trauma (n=17); or did not include online or mobile interventions (n=93) targeting substance misuse (n=23). A total of 159 records were therefore included in this review. These 159 records reflected 141 distinct studies, including 125 distinct interventions.

### Study Characteristics

The study characteristics are provided in Table 1. In-depth characteristics of the included studies are described in Multimedia Appendix 2 and Multimedia Appendix 3, and the intervention characteristics are shown in Multimedia Appendix 4. The majority of studies were conducted in the United States (94/161, 58.4%). The other locations were the European Union (38/161, 23.6%), United Kingdom (7/161, 4.3%), Australia and New Zealand (11/161, 6.8%), Canada (7/161, 4.3%), and others (<4%). Studies included one to six conditions (mean 2.3, median 2), with the majority (n=149) randomizing participants to conditions. The majority of studies included control conditions (n=115), including treatment as usual (n=31), assessment only (n=20), waitlist (n=12), and other control conditions specifically relevant to the research question. Sample sizes ranged from 13 to 4165 (mean 453, median 217), and sample types included clinical (50/159, 31.4%), community (56/159, 35.2%), college/university (40/159, 25.2%), and veteran samples (7/159, 4.4%) and others (5/159, 3.1%). The mean age of the participants ranged from 18 to 53 years (mean 31.83, median 34). The majority of studies were focused on alcohol use, risks, and/or harms (109/159, 68.5%), although a substantial minority focused on multiple substances or any substance (28/159, 17.6%), cannabis specifically (12/159, 7.5%), opioids specifically (4/159, 2.5%), or other specific substances (<3% each).

**Table 1:** Characteristics of the included studies (N=159).

Parameters and characteristics				Studies, n (%)^a^
**Study metadata**				
	**Study design**			
		Randomized controlled trial	122 (76.3%)	
		Secondary analyses	18 (11.3%)	
		Single-arm studies	19 (11.9%)	
	**Location^b^**			
		United States	94 (58.4%)	
		Canada	7 (4.4%)	
		European Union	38 (23.6%)	
		United Kingdom	7 (4.4%)	
		South America	1 (0.6%)	
		Australia and New Zealand	11 (6.8%)	
		Asia	3 (1.9%)	
**Population characteristics**				
	**Sample size**			
		≤100	41 (25.6%)	
		101-500	66 (41.3%)	
		501-1000	39 (24.4%)	
		>1000	14 (8.8%)	
	Mean age (years)^c^		31.83	
	**Percentage women/female**			
		≤10%	3 (1.9%)	
		11%-50%	95 (59.4%)	
		51%-99%	54 (33.8%)	
		100%	8 (5.0%)	
**Study characteristics**				
	**Target substance**			
		Alcohol	110 (69.2%)	
		Cannabis	12 (7.6%)	
		Opioids	4 (2.5%)	
		Any substance	28 (17.6%)	
		Other substances	5 (3.1%)	
	**Conducted sex- and gender-based analyses**			
		Yes	27 (17.0%)	
		No	123 (77.3%)	
		N/A^d^	9 (5.7%)	
	**Assessed gender**			
		Yes	19 (12.0%)	
		No	140 (88.0%)	
	**Assessed trauma**			
		Yes	13 (9.1%)	
		No	147 (90.9%)	
**Intervention characteristics (n=190)**				
	**Language**			
		English	150 (79.0%)	
		Spanish	3 (1.6%)	
		Swedish	15 (7.9%)	
		German	6 (3.2%)	
		Norwegian	2 (1.1%)	
		Multiple	6 (3.2%)	
		Other	5 (2.6%)	
	**Nature of the intervention**			
		Single-session intervention	68 (35.8%)	
		Multisession intervention	122 (64.2%)	
	**Mode of delivery**			
		Mobile (app)	27 (14.2%)	
		Mobile (text)	13 (6.8%)	
		Online	148 (77.9%)	
		Combined (online + mobile)	2 (1.1%)	

^a^Percentages were rounded and may not sum to 100.

^b^Numbers do not add up to 159 as two studies were conducted in multiple locations.

^c^Mean age was not reported in nine studies.

^d^N/A: not applicable.

### Intervention Characteristics

Digital health resources included multisession interventions (122/190, 64.2%) with multiple components or modules, such as screening and assessment, motivational enhancement, psychoeducation, and cognitive and behavioral skills building. Multisession interventions were available online (82/122, 67.2%), on mobile devices (mobile apps [25/122, 20.5%] and text [13/122, 10.7%]), and in a combination of both online and mobile methods (2/122, 1.6%). A substantial minority comprised single-session interventions (68/190, 35.8%), which were primarily available online (67/68, 99%) rather than on mobile devices (mobile apps [1/68, 2%]). There were no single-session interventions that were provided over text or that comprised a combination of both online and mobile methods. Each of these broad categories of digital health resources will be discussed in turn below. The digital health intervention duration ranged from one session (n=68) to 12 months, with other frequent durations including 4 weeks (n=7), 8 weeks (n=10), and 12 weeks (n=28). A substantial minority did not report or include a follow-up period (n=35). Follow-up durations ranged from 2 weeks to 2 years, with the most frequent periods including 1 month (n=37), 3 months (n=49), and 6 months (n=52). Overall, interventions were primarily in the English language (150/190, 78.9%), although others were available in multiple languages (6/190, 3.2%) or other specific languages such as Swedish (15/190, 7.9%) and others (German [6/190, 3.2%], Norwegian [2/190, 1.1%], Spanish [3/190, 1.6%], other [5/190, 2.6%]; all <4%). In line with the study focus as reviewed above, the majority of interventions themselves targeted alcohol use, risks, and/or harms (95/124, 76.6%) in their content, with a substantial minority including a treatment targeting multiple substances or any substance (15/124, 12.1%), cannabis specifically (11/124, 8.9%), or other specific substances or substance combinations (<2% each).

### Study Quality or Bias

**Figure 2:**
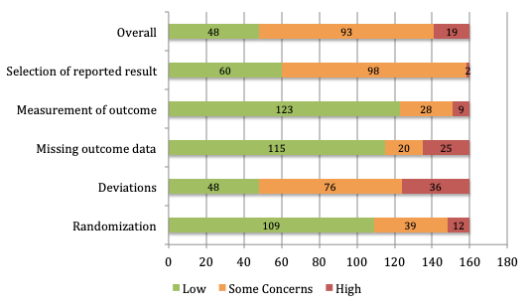
Risk of bias distributions.

Overall, the majority of studies were found to exhibit features associated with some concerns of bias (93/160, 58.1%), with 11.9% (19/160) associated with high bias and 30.0% (48/160) associated with low bias, according to the Cochrane Risk of Bias Tool (Figure 2). More specifically, 68.1% (109/160) of studies were evaluated to have low bias associated with the randomization process (Domain 1), suggesting that adequate processes were put in place within these studies to minimize issues with randomization. The 39 studies associated with some concerns reported limited information on randomization methods or the concealment of assigned interventions, while the 12 with high bias did not randomize participants.

Domain 2 examined the risk of bias due to deviations from the intended interventions. Approximately 48% (76/160, 47.5%) of studies were evaluated as having some concerns of bias in this domain, mostly due to the use of analyses that were not appropriate to estimate the effect of assignment to the intervention, or the lack of adequate information regarding deviations from the planned protocol. The 22.5% (36/160) of studies with high bias tended to have issues regarding both of these points, whereas the 30.0% (48/160) of studies with low bias used intention-to-treat analyses to estimate the effect of assignment and had adequate blinding measures in place.

Domain 3 examined risk of bias due to missing outcome data, with 71.9% (115/160) of studies being evaluated as having low bias. Low bias in this domain signified that outcome data were available for nearly all participants or that adequate measures were put in place to evaluate bias due to missing outcome data. The 12.5% (20/160) of studies with some concerns were evaluated as such if it was possible that the results were biased by missing outcome data, such that study withdrawal occurred due to participants’ health status, whereas the 15.6% (25/160) of studies with high bias were evaluated as such if it was *likely* that study withdrawal occurred for this reason.

Domain 4 examined risk of bias in measurement of the outcome variables. Approximately 77% (123/160, 76.9%) of studies were evaluated to have low bias in this section, due to appropriate outcome measures being used and appropriate blinding if outcomes were assessed by outcome assessors or participant blinding if outcomes were assessed using self-report measures. The 17.5% (28/160) of studies evaluated as having some concerns in this domain were characterized by some likelihood that measures of outcomes could have been influenced by the intervention received, and the 5.6% (9/160) of studies rated as having high bias in this domain were found to have inadequate information in this regard.

Domain 5 examined risk of bias in the selection of the reported results. Approximately 61% (98/160, 61.3%) of studies were rated as having some concerns in this domain, particularly due to lack of prespecified analysis plans. The 37.5% (60/160) of studies with low risk of bias were found to have prespecified analysis plans and report all outcome measures and analyses in accordance with these plans. The 1.3% (2/160) of studies evaluated as having high risk of bias were found to be potentially selective in reporting outcome measurements and analyses, based on the results. All domains were coded according to a standardized scoring algorithm. Detailed information regarding the risk of bias for each study is presented in Multimedia Appendix 5. See Multimedia Appendix 6 for the references for all studies.

### Study Outcomes

Overall, studies concluded that digital health resources for substance use or associated harms were efficacious or effective (155/190, 81.6%). The proportion of participants who identified as female or women ranged from 7% to 100% (mean 48%, median 46%; six studies were below 20%, but were eligible as over 20% endorsed trauma). In the vast majority of cases, participants identified as female, as only 16 studies explicitly assessed gender identity. Many studies appeared to use the terms sex and gender interchangeably (n=41). For example, indicating that gender was assessed (rather than sex) and specifying that reported genders were female and male. Sex- and gender-based analyses were conducted in only 17.0% (27/159) of studies, with 77.4% (123/159) of studies not conducting such analyses and 5.7% (9/159) not applicable (ie, no females or women were included in the sample, or the sample included only females or women). Thus, although digital health resources were found to be efficacious or effective in general, this was quantitatively confirmed for females or women in only 13.7% (26/190) of studies, with 81.1% (154/190) of studies not reporting relevant analyses and 5.3% (10/190) finding that the intervention was not effective for female or women participants. Only 13 of the studies reported that at least 20% of participants had a trauma history, and only seven of these reported that at least 20% of participants were female or women, *and* reported trauma (ie, six studies included at least 20% of participants with a trauma history, but less than 20% were female or women; these studies were nevertheless retained to present the nature of the evidence for those who have been exposed to violence or trauma). These studies included two studies of single-session interventions as follows: BSAFER (developed for any substance [32]; demonstrated effectiveness at a 3-month follow-up in a small sample) and VetChange (developed for alcohol [33]; demonstrated effectiveness over 1, 3, and 6 months). Two studies evaluated a mobile app (A-CHESS) developed for any substance and delivered over 6 to 8 months, which demonstrated effectiveness in an entirely female sample [34], as well as a mixed sample [35], although sex- or gender-based analyses were not conducted in the latter. Another study evaluated a mobile text message intervention for alcohol in young adults following emergency room treatment, with improvements at a 3-month follow-up [36]. Finally, two widely investigated interventions (CBT4CBT and TES) developed for any substance and delivered over 12 weeks were evaluated in samples involving females and trauma, and although both interventions were effective, sex- or gender-based analyses were not conducted [37,38].

A total of 122 study conditions comprised online multisession interventions, primarily targeting alcohol (n=53) or any substance (n=19), although more targeted interventions for cannabis and opioids were present as well. These interventions included both openly available and commercial products, which varied in their provision of screening, assessment, or monitoring; however, most included psychoeducation, goal setting, cognitive and behavioral skills training, and links to resources. Primary outcomes were most frequently substance consumption, although substance use harms or substance use disorder symptoms were also included. Overall, 87% (73/84) of these relatively intensive interventions exhibited acute impacts on primary outcomes following up to 8 or 12 weeks of treatment; in some cases, these were retained in subsequent follow-up assessments.

Mobile interventions included both apps (n=27) and text-based messaging interventions (n=13). Mobile apps targeted alcohol (n=21) and any substance (n=4) or cannabis (n=3), and 87% (73/84) of these apps demonstrated improvements in the primary outcomes after approximately 4 to 12 weeks of use. Text-based messaging interventions targeted alcohol (n=9) or cannabis (n=1), and 85% (11/13) demonstrated benefits following 2 to 12 weeks of use.

A total of 67 study conditions evaluated brief interventions, which were primarily delivered online (n=66) as compared to via a mobile device (n=1). The majority of these brief interventions addressed harmful or risky alcohol use, with only a small number addressing general drug use (n=10) or cannabis use (n=3). These brief interventions frequently took the form of noncommercial programs that provided initial screening and personalized normative feedback, as well as psychoeducation and resources. The primary outcome was most frequently substance consumption, primarily quantity or frequency (eg, number of standard alcoholic drinks per week and binge or heavy drinking frequency). Approximately 64% (43/67) of these brief interventions did exhibit short-term impacts on the primary outcomes.

## Discussion

### Principal Results

The empirical investigations of the efficacy or effectiveness of digital health resources for adults who identify as female or women, or who report a history of trauma, appear to be principally conducted in the United States and Europe, with the majority in the English language. These investigations and the interventions evaluated predominantly focused on alcohol use or associated harms/risks, although a substantial minority of investigations was broadly applicable to substance use in general. The majority of studies randomized participants to study conditions, with a range of active and control conditions evident across studies. Similar to other reviews of psychosocial interventions, a substantial proportion of investigations was judged to have some concerns associated with bias, primarily related to participant or assessor blinding, lack of intent-to-treat analysis, or lack of a reported prespecified or registered analytical plan. Lower bias was evident regarding randomization, missing data, and outcome measurement.

The digital health resources evaluated included multisession and brief (ie, single) session interventions, with a wide range of therapeutic elements. Across all interventions, the primary outcome was most frequently substance use quantity and frequency. More intensive online and mobile interventions, frequently several months or more in duration and including numerous therapeutic components, exhibited moderate to strong effects in the vast majority of studies. Brief interventions, which consisted of a single session of varied duration (but most commonly less than 1 hour), demonstrated efficacy in most studies, although it was notable that these effects decreased over longer follow-ups in many studies.

Overall, studies that included a substantial proportion of adults who identified as female or women concluded that digital health resources for substance use or associated harms were efficacious or effective (155/190, 81.6%). A minimum threshold of 20% of the sample identifying as female or women, or endorsing trauma, was implemented to ensure the relevance of evidence reviewed to the research question. This eligibility requirement resulted in the exclusion of a limited number of records (n=17), which shared many of the study and intervention features described above. Notably, in many contexts, a much higher proportion would be required to conduct sex- or gender-based analyses and to support generalizability to our target populations. In fact, the majority of studies did include 40% or more of participants who were female or women, with larger proportions more common in community and trainee samples. Yet, most investigations did not assess gender identity, and many used sex and gender terms interchangeably. Further, sex- or gender-based analyses were not conducted in the majority of studies (n=113); thus, direct or quantitative evidence for the efficacy or effectiveness of interventions in females or women specifically is weak.

Evidence for adults reporting a history of trauma was even more limited. Only 13 studies were found that met this liberal inclusion criterion, and even then, the association between trauma history and clinical outcomes was not evaluated. There is a critical need to assess and report trauma in the evaluation of digital health resources in this context to identify those most likely to be of benefit to adults with a trauma history. Of note, the current results appear unlikely to be the result of lower access to individuals with past or current experiences of violence and trauma. Among women presenting to treatment, significantly higher rates of sexual abuse have been observed in comparison to community samples of women meeting criteria for the diagnosis of substance use disorders, suggesting that experiences of trauma may play a role in the process of treatment initiation [10,39]. In fact, 61% of people in a population of treatment-seeking men and women specifically cited the experience of a recent traumatic event as the reason for seeking treatment for their substance misuse, demonstrating a clear need for a trauma-focused approach [39].

### Comparison With Prior Work

Similar to the current investigation, previous systematic reviews and meta-analyses have highlighted the large number of digital interventions for alcohol, with a preponderance of brief interventions with small immediate benefits but low evidence for longer-term clinically significant effects [40]. Evidence for digital interventions for other substances is promising but more limited [41].

The most recent and focused investigation of digital health resources for women with substance use disorders focused on the childbearing age. Notably, the current broader synthesis noted that, in fact, there appears to be a dearth of studies in older adult samples as well as studies in other important groups. For example, studies in samples across the lifespan, across racial backgrounds, and with other important social determinants of health and those who face barriers to care (eg, rural communities, homeless or houseless individuals, forensic samples, and adults of varied physical and mental abilities) are critical to conduct. Thus, although a range of sample types was evident in the current review, future research would benefit from extending across the lifespan and including other types of samples with more varied demographic and clinical features.

### Limitations

This investigation focused on a specific category of digital health resources, which necessarily limits its scope. The consideration of virtual psychotherapy, digital recovery support networks, and other forms of resources would be a valuable extension of this work. Similarly, this investigation focused on adults reporting or exhibiting substance use risks or harms, and interventions targeting substance use or associated risks or harms, which would preclude larger-scale population health interventions targeting a broader range of lower-risk individuals as well as interventions with lifestyle or health/wellness foci. Focused reviews of these broader groups and interventions would benefit a range of stakeholders. This review focused on adults who identify as female or women and neglected other sex and gender groups. Thus, increased attention to treatment outcomes across the gender continuum is needed. Finally, the incorporation of other key identity features, particularly those related to race, culture, and ethnicity, is critical to examine how the intersections of these different components of identity are linked to treatment outcomes. Very limited research in this area has been conducted to date, highlighting this key gap.

### Conclusions

This project represents a synthesis of available evidence for digital health resources for adults who identify as female or women with substance use concerns. Although substance use has been increasing in these individuals, adults who identify as female or women are underrepresented in in-person clinical services and exhibit unique treatment barriers, preferences, and needs. Importantly, trauma is elevated in this group, highlighting the clinical priority of interventions that are sensitive to not only gender-specific psychoeducation and skills building, but also trauma-informed approaches. Although this synthesis simultaneously provides promising support for the therapeutic benefit of digital health resources for this priority population, it also highlights critical clinical and research priorities. Increased assessments of both sex and gender identities, and the implementation of sex- and gender-based analyses are critical in future empirical investigations of digital health resources. Increased integration of trauma and other key participant features is also needed to contribute to the further development of these interventions. Trauma, intersectionality, and key social determinants of health are critical to understand not only the value of these resources but also how to successfully implement them in varied geographical regions and health systems.

## Appendix

Multimedia Appendix 1 Search strategy for MEDLINE.

Multimedia Appendix 2 Study demographic characteristics.

Multimedia Appendix 3 Study design characteristics.

Multimedia Appendix 4 Intervention characteristics.

Multimedia Appendix 5 Risk of bias.

Multimedia Appendix 6 References for articles included in the scoping review.
