# Supplementary material for: Benefits of Digital Health Resources for Substance Use Concerns in Women: Scoping Review
Source: JMIR Ment Health. 2021 Jun 7;8(6):e25952. doi: 10.2196/25952 (PMC8218208; doi:10.2196/25952)
Supplement: Multimedia Appendix 1 [file mental_v8i6e25952_app1.docx]

Multimedia Appendix 1: Search Strategy for Medline

Database: Ovid MEDLINE(R) ALL <1946 to June 29, 2020>

Search Strategy:

1. exp Substance-Related Disorders/

2. ((drug* or substance*) adj2 (abus* or addict* or depend* or misus* or "use" or dependen* or disorder*)).mp.

3. ((alcohol* adj2 (abus* or addict* or depend* or misus* or "use" or dependen* or disorder*)) or alcoholic* or alcoholism).mp.

4. exp Cannabis/

5. exp Hallucinogens/

6. exp Phencyclidine/

7. exp Analgesics, Opioid/

8. exp "Hypnotics and Sedatives"/

9. exp Central Nervous System Stimulants/

10. exp Amphetamine/

11. exp Anti-Anxiety Agents/

12. exp Illicit Drugs/

13. exp Cocaine/

14. (abus* or addict* or depend* or disorder* or harm* or problem$ or misus* or "use").mp.

15. (((alcohol* or drug$ or cannab* or marijuana$ or marihuana$ or bhang$ or ganja$ or hashish$ or hemp$ or hallucinogen* or inhal* or phencyclidine$ or opioid$ or opiate$ or hypnotic$ or sedativ* or anxiolytic$ or (anti-anxiet* adj (drug$ or agent$)) or depressant$ or stimulant$ or amphetamine$ or cocaine$ or analgesic* or heroin) adj2 (abus* or addict* or depend* or disorder* or harm* or problem* or misus* or non-medical* or nonmedical* or "use")) or (((street drug$ or heroin or IV) adj3 drug$) or (intravenous adj3 drug$)) or (illicit drug$ or "recreational drug" or "recreational drugs" or benzo$)).mp.

16. or/1-3 [i) substance use disorders]

17. ((or/4-13) and 14) or 15 [ii) substance use disorders]

18. exp Internet/

19. Mobile Applications/

20. Technology/

21. Internet-Based Intervention/

22. Computers/

23. exp Computers, Handheld/

24. user-computer interface/

25. Online Systems/

26. Self Care/

27. (intervention$ or self-help* or selfhelp* or self-car* or selfcar* or platform$ or psychiatr* or pscyhos?s$ or psychotherap* or psycho-educat* or psychoeducat* or treatment$ or mhealth or m-health or (mobile adj health)).mp.

28. (((app or apps or computer* or digital* or ehealth or e-health or electronic or internet* or Mhealth or "m health" or mobile or online or on-line or smartphone or smart-phone or technology or web* or virtual*) adj3 (intervention$ or module* or self-help* or selfhelp* or self-car* or selfcar* or platform$ or program* or psychiatr* or pscyhos?s$ or psycho-educat* or psychoeducat* or resource$ or treatment$)) or (mhealth or m-health or (mobile adj health))).mp.

29. ((or/18-25) and (or/26-27)) or 28 [digital health interventions]

30. Female/ and (exp Adult/ or adult$.mp.)

31. female$.mp. and (exp Adult/ or adult$.mp.)

32. Women/

33. battered women/

34. pregnant women/

35. Mothers/

36. (wom?n$ or mother* or maternal* or pregnan*).mp.

37. exp "Trauma and Stressor Related Disorders"/

38. exp Violence/

39. exp Sex Offenses/

40. (trauma* or abus* or assault* or post-trauma* or postrauma* or PTSD or rape or raped or violen* or crime$).mp.

41. exp crime victims/

42. exp Survivors/

43. (sufferer$ or surviv* or victim$).mp.

44. or/30-36 [women]

45. or/37-40 [trauma concept]

46. or/41-43 [survivor concept]

47. 45 and 46 [trauma survivors]

48. or/16-17 [substance use disorders]

49. 29 [digital health interventions]

50. 44 or 47 [women or trauma survivors]

51. 48 and 49 and 50

52. (comment or editorial or interview or news or newspaper article).pt.

53. 51 not 52

54. limit 53 to yr="2014 -Current"
