# Supplementary material for: Benefits of Digital Health Resources for Substance Use Concerns in Women: Scoping Review
Source: JMIR Ment Health. 2021 Jun 7;8(6):e25952. doi: 10.2196/25952 (PMC8218208; doi:10.2196/25952)
Supplement: Multimedia Appendix 2 [file mental_v8i6e25952_app2.docx]

Multimedia Appendix 2: Study demographic characteristics

| Author | Year | Location | Sample Type | Target Substance | Overall N | % Women/ | % Trauma | Mean Age |
| --- | --- | --- | --- | --- | --- | --- | --- | --- |
|  |  |  |  |  |  | Female |  |  |
| Acosta | 2017 | USA | Veterans | Any Substance | 162 | 7% | 79% | 34.0 |
| Acosta | 2012 | USA | Clinical | Opioids | 160 | 25% | NR | 40.7 |
| Acuff | 2019 | USA | College/University | Alcohol | 608 | 54% | NR | 18.6 |
| Aharonovich | 2017 | USA | Community | Any Substance | 47 | 23% | NR | 50.9 |
| Albertella | 2019 | USA | Community | Cannabis | 111 | 32% | NR | 26.7 |
| Baldin | 2018 | South America | Community | Alcohol | 465 | 36% | NR | 24.7 |
| Barrio | 2017 | EU | Clinical | Alcohol | 24 | 50% | NR | 48.0 |
| Berman | 2019 | EU | College/University | Alcohol | 2166 | 68% | NR | 25.8 |
| Berman | 2020 | EU | Community | Alcohol | 89 | 70% | NR | 48.9 |
| Bertholet | 2019 | USA | Community | Alcohol | 977 | 46% | NR | 34.2 |
|  |  | Canada |  |  |  |  |  |  |
| Bertholet | 2017 | Canada | Community | Alcohol | 130 | 48% | NR | 32.8 |
|  |  | EU |  |  |  |  |  |  |
| Blankers | 2011 | Netherlands | Community | Alcohol | 205 | 51% | NR | 42.2 |
| Blankers | 2013 | Netherlands | Clinical | Alcohol | 205 | 51% | NR | 41.5 |
| Bo | 2018 | EU | Community | Alcohol | 434 | 56% | NR | 47.5 |
| Bock | 2016 | USA | College/University | Alcohol | 60 | 62% | NR | 21.8 |
| Boyle | 2018 | USA | College/University | Alcohol | 141 | 51% | NR | NR |
| Brendryen | 2017 | EU | Other | Alcohol | 85 | 52% | NR | 43.0 |
| Brendryen | 2014 | EU | Community | Alcohol | 244 | 33% | NR | 38.0 |
| Brief | 2018 | USA | Veterans | Alcohol | 523 | 14% | 64% | 31.9 |
| Brief | 2013 | USA | Veterans | Alcohol | 600 | 13% | 61% | 32.0 |
| Brooks | 2010 | USA | Clinical | Cocaine | 26 | 50% | NR | 43.0 |
| Budney | 2011 | USA | Community | Cannabis | 38 | 47% | NR | 32.8 |
| Budney | 2015 | USA | Community | Cannabis | 75 | 44% | NR | 35.1 |
| Campbell | 2015 | USA | Clinical | Any Substance | 506 | 38% | NR | 34.9 |
| Campbell | 2017 | USA | Clinical | Any Substance | 507 | 38% | NR | 34.9 |
| Campbell | 2014 | USA | Clinical | Any Substance | 507 | 38% | NR | 34.9 |
| Campbell | 2016 | USA | Community | Alcohol | 188 | 61% | NR | 44.3 |
| Carey | 2017 | USA | College/University | Alcohol | 326 | 61% | NR | 20.9 |
| Carey | 2011 | USA | College/University | Alcohol | 677 | 36% | NR | 19.0 |
| Carra | 2016 | EU | Community | Alcohol | 590 | 52% | NR | 20.6 |
| Chiauzzi | 2005 | USA | College/University | Alcohol | 265 | 54% | NR | 19.9 |
| Choo | 2016 | USA | Clinical | Any Substance | 40 | 100% | 45% | 25.0 |
| Christensen | 2014 | USA | Community | Opioids | 170 | 46% | NR | 34.4 |
| Chung | 2016 | USA | Clinical | Alcohol | 765 | 65% | NR | 22.0 |
| Cochrane | 2015 | USA | Clinical | Any Substance | 507 | 38% | NR | 34.9 |
| Collins | 2014 | USA | College/University | Alcohol | 724 | 56% | NR | 20.8 |
| Copeland | 2017 | Australia & NZ | Community | Cannabis | 287 | 38% | NR | 26.0^a^ |
| Crane | 2018 | UK | Community | Alcohol | 672 | 56% | NR | 39.2 |
| Cunningham | 2017 | Canada | Community | Alcohol | 490 | 50% | NR | 37.6 |
| Cunningham | 2012 | Canada | Community | Alcohol | 170 | 41% | NR | 45.0 |
| Cunningham | 2010 | Canada | Community | Alcohol | 185 | 47% | NR | 40.1 |
| Cunningham | 2012 | Canada | College/University | Alcohol | 425 | 47% | NR | 22.6 |
| Cunningham | 2009 | Canada | Community | Alcohol | 185 | 47% | NR | 40.1 |
| Deady | 2016 | Australia & NZ | Community | Alcohol | 104 | 60% | NR | 21.7 |
| Delrahim-Howlett | 2011 | USA | Clinical | Alcohol | 150 | 100% | NR | 26.3 |
| DeMartini | 2018 | USA | Clinical | Alcohol | 15 | 27% | NR | 50.8 |
| Doumas | 2009 | USA | College/University | Alcohol | 76 | 28% | NR | 19.2 |
| Dulin | 2017 | USA | Community | Alcohol | 28 | 42% | NR | 33.9 |
| Dulin | 2014 | USA | Community | Alcohol | 28 | 46% | NR | 33.6 |
| Dunn | 2020 | USA | College/ University | Alcohol | 121 | 33% | NR | 19.4 |
| Elison | 2015a | UK | Clinical | Alcohol | 300 | 45% | NR | 41.9 |
| Elison | 2015b | UK | Other | Any Substance | 393 | 43% | NR | 42.4 |
| Elison | 2017 | UK | Clinical | Any Substance | 2311 | 45% | NR | 42.2 |
| Fazzino | 2016 | USA | College/University | Alcohol | 856 | 70% | NR | 20.0 |
| Finfgeld-Connett | 2008 | USA | Community | Alcohol | 67 | 100% | NR | 50.0 |
| Gajecki | 2017 | EU | College/University | Alcohol | 330 | 69% | NR | 25.4 |
| Gajecki | 2014 | EU | College/University | Alcohol | 1929 | 52% | NR | 24.7 |
| Geisner | 2015 | USA | College/University | Alcohol | 311 | 62% | NR | 20.1 |
| Gilmore | 2016 | USA | College/University | Alcohol | 264 | 100% | NR | 18.8 |
| Gilmore | 2018 | USA | College/University | Alcohol | 264 | 100% | NR | 18.8 |
| Gilmore | 2015 | USA | College/University | Alcohol | 207 | 100% | NR | 18.8 |
| Glass | 2017 | USA | Clinical | Alcohol | 349 | 39% | NR | 38.3 |
| Gonzales | 2014 | USA | Clinical | Any Substance | 81 | 27% | NR | 20.4 |
| Gonzales-Castaneda | 2019 | USA | Clinical | Any Substance | 80 | 29% | NR | 20.7 |
| Gonzalez | 2015 | USA | Community | Alcohol | 54 | 42% | NR | 34.0 |
| Guarino | 2016 | USA | Clinical | Opioids | 50 | 20% | NR | 42.2 |
| Guillemont | 2017 | EU | Community | Alcohol | 1147 | 46% | NR | NR |
| Gustafson | 2014 | USA | Clinical | Alcohol | 349 | 39% | 53% | 38.4 |
| Haskins | 2017 | USA | Clinical | Alcohol | 212 | 62% | NR | 38.1 |
| Haug | 2015 | EU | Clinical | Alcohol | 50 | 24% | NR | 47.1 |
| Hansen | 2012 | Denmark | Community | Alcohol | 1380 | 45% | NR | 58.0^a^ |
| Hester | 2011 | USA | Community | Alcohol | 75 | 56% | NR | 50.0 |
| Hester | 2012 | USA | College/University | Alcohol | 144 | 38% | NR | 20.4 |
|  |  |  |  | Alcohol | 82 | 44% | NR | 20.15 |
| Hester | 2009 | USA | College/University | Alcohol | 84 | 56% | NR | 50.0 |
| Hester | 2013 | USA | Community | Alcohol | 189 | 60% | NR | 44.3 |
| Hester | 2005 | USA | Community | Alcohol | 61 | 48% | NR | 45.7 |
| Hunter | 2017 | EU | Clinical | Alcohol | 763 | 38% | NR | 29.5 ^a^ |
| Ingersoll | 2018 | USA | Community | Alcohol | 75 | 100% | NR | 27.8 |
| Jo S-J | 2019 | Asia | Community | Alcohol | 1496 | 48% | NR | 30.0 ^a^ |
| Johansson | 2017 | EU | Community | Alcohol | 4165 | 52% | NR | 41.9 |
| Johnston | 2019 | USA | Clinical | Any Substance | 180 | 100% | 70% | 27.9 |
| Jonas | 2018 | EU | Community | Cannabis | 534 | 34% | NR | 27.5 |
| Jonas | 2019 | EU | Community | Cannabis | 534 | 34% | NR | 27.5 |
| Kazemi | 2020 | USA | College/University | Alcohol | 379 | 55% | NR | 19.9 |
| Khadjesari | 2014 | UK | Other | Alcohol | 736 | 25% | NR | 48.0 ^a^ |
| Kiluk | 2016 | USA | Clinical | Alcohol | 68 | 35% | NR | 42.7 |
| Kiluk | 2018 | USA | Clinical | Any Substance | 137 | 25% | NR | 35.9 |
| Kim | 2016 | USA | Clinical | Opioids | 160 | 25% | NR | 40.7 |
| Klein | 2013 | USA | Clinical | Any substance | 1682 | 43% | NR | 41.8 |
| Klein | 2012 | USA | Clinical | Any substance | 1124 | 45% | NR | 42.1 |
| Kypri | 2009 | Australia & NZ | College/University | Alcohol | 2435 | 45% | NR | 19.7 |
| Kypri | 2008 | Australia & NZ | Clinical | Alcohol | 576 | 52% | NR | 20.1 |
| Kypri | 2013 | Australia & NZ | College/University | Alcohol | 1789 | 66% | NR | 20.2 |
| Kypri | 2004 | Australia & NZ | College/University | Alcohol | 104 | 50% | NR | 20.2 |
| Leeman | 2016 | USA | College/University | Alcohol | 208 | 62% | NR | 19.9 |
| Levesque | 2017 | USA | Clinical | Any Substance | 437 | 38% | NR | 35.3 |
| Lewis | 2019 | USA | Community | Alcohol | 402 | 54% | NR | 22.4 |
| Liang | 2018 | Asia | Other | Any Substance | 75 | 29% | NR | 41.6 |
| Linowski | 2016 | USA | College/University | Alcohol | 346 | 40% | NR | 19.0 |
| Livingston | 2020 | USA | Veterans | Alcohol | 222 | 22% | 55% | 36.0 |
| Mariano | 2019 | USA | Clinical | Any Substance | 507 | 38% | NR | 34.5 |
| Marsch | 2014 | USA | Clinical | Opioids | 160 | 25% | NR | 40.7 |
| Mason | 2014 | USA | College/University | Alcohol | 18 | 56% | NR | 19.2 |
| Mason | 2020 | USA | Community | Cannabis | 96 | 43% | NR | 20.3 |
| Miller | 2018 | USA | Veterans | Alcohol | 571 | 17% | 23% | 28.9 |
| Muench | 2017 | USA | Community | Alcohol | 152 | 75% | NR | 43.2 |
| Murphy | 2010 | USA | College/University | Alcohol | 133 | 50% | NR | 18.6 |
| Murphy | 2015 | USA | College/University | Alcohol | 133 | 50% | NR | 18.6 |
| Murray | 2012 | UK | Clinical | Alcohol | 19 | 53% | NR | 42.0 |
| Neighbors | 2010 | USA | College/University | Alcohol | 818 | 58% | NR | 18.2 |
| Osilla | 2015 | USA | Other | Alcohol | 159 | 35% | NR | 30.0 |
| Paris | 2018 | USA | Clinical | Any Substance | 92 | 33% | 47% | 43.0 |
| Pedersen | 2017 | USA | Veterans | Alcohol | 793 | 17% | 39% | 28.9 |
| Possemato | 2019 | USA | Veterans | Alcohol | 30 | 7% | 60% | 39.0 |
| Riper | 2008 | EU | Community | Alcohol | 261 | 49% | NR | 46.1 |
| Rooke | 2014 | Australia & NZ | Community | Cannabis | 390 | 39% | NR | 31.1 |
| Schaub | 2019 | EU | Community | Cocaine | 311 | 27% | NR | 33.0 |
| Schaub | 2012 | EU | Community | Cocaine | 196 | 22% | NR | 34.2 |
| Schulz | 2013 | EU | Community | Alcohol | 448 | 44% | NR | 41.7 |
| Sharpe | 2019 | Australia & NZ | Clinical | Alcohol | 598 | 29% | NR | 34.0 |
| Sharpe | 2018 | Australia & NZ | Clinical | Alcohol | 598 | 29% | NR | 34.0 |
| Shrier | 2014 | USA | Clinical | Cannabis | 27 | 70% | NR | 19.0 |
| Shulman | 2018 | USA | Clinical | Any Substance | 507 | 38% | NR | 34.9 |
| Sinadinovic | 2014a | EU | Community | Any Substance | 633 | 55% | NR | 44.0 |
| Sinadinovic | 2014 b | EU | Community | Any Substance | 202 | 48% | NR | 33.2 |
| Sinadinovic | 2020 | EU | Community | Cannabis | 303 | 33% | NR | 27.4 |
| Sinadinovic | 2012 | EU | Community | Any substance | 202 | 47% | NR | 33.2 |
| Steers | 2016 | USA | College/University | Alcohol | 176 | 82% | NR | 23.3 |
| Suffoletto | 2014 | USA | Clinical | Alcohol | 765 | 65% | NR | 21.9 |
| Suffoletto | 2015 | USA | Clinical | Alcohol | 765 | 65% | NR | 21.9 |
| Suffoletto | 2020 | USA | Clinical | Alcohol | 127 | 73% | NR | 21.8 |
| Suffoletto | 2012 | USA | Clinical | Alcohol | 45 | 54% | 28% | 21.0 |
| Sundstrom | 2016 | EU | Community | Alcohol | 80 | 60% | NR | 42.3 |
| Sundstrom | 2020 | EU | Community | Alcohol | 166 | 51% | NR | 52.9 |
| Sundstrom | 2017 | EU | Community | Alcohol | 13 | 69% | NR | 49.5 |
| Susukida | 2018 | USA | Clinical | Any Substance | 507 | 38% | NR | NR |
| Tahaney | 2017 | USA | College/University | Alcohol | 111 | 72% | NR | 19.0 |
| Tait | 2015 | Australia & NZ | Community | Other | 160 | 24% | NR | 22.4 |
| Tait | 2019 | Australia & NZ | Community | Alcohol | 793 | 71% | NR | 41.0 |
| Takano | 2020 | Asia | Clinical | Any Substance | 48 | 32% | NR | 38.0 |
| Teeters | 2018 | USA | College/University | Alcohol | 84 | 64% | NR | 22.5 |
| Tensil | 2013 | EU | Community | Alcohol | 595 | 39% | NR | 29.4 |
| Tetrault | 2020 | USA | Clinical | Other | 58 | 43% | NR | 43.5 |
| Tofighi | 2016 | USA | Clinical | Any Substance | 507 | 38% | 21% | 34.9 |
| Vaezazizi | 2019 | USA | Clinical | Any Substance | 507 | 38% | NR | 35.1 |
| Van Lettow | 2015 | EU | Community | Alcohol | 2634 | 49% | NR | 37.0 |
| Voogt | 2014 | EU | College/University | Alcohol | 907 | 40% | NR | 20.8 |
| Voogt | 2013a | EU | College/University | Alcohol | 913 | 40% | NR | 20.8 |
| Voogt | 2013b | EU | College/University | Alcohol | 913 | 40% | NR | 20.8 |
| Wallace | 2017 | EU | Clinical | Alcohol | 763 | 38% | NR | 49.5 ^a^ |
| Walukevich-Dienst | 2019 | EU | College/University | Cannabis | 204 | 77% | NR | 19.8 |
| Walukevich-Dienst | 2020 | USA | College/University | Cannabis | 204 | 77% | NR | 19.8 |
| Ward | 2019 | UK | Clinical | Alcohol | 1937 | 46% | NR | 44.1 |
| Wilks | 2018 | USA | Community | Alcohol | 59 | 70% | NR | 38.0 |
| Wilson | 2015 | USA | Clinical | Any Substance | 95 | 50% | NR | NR |
| Witkiewitz | 2014 | USA | College/University | Alcohol | 94 | 28% | NR | 20.5 |
| Young | 2019 | USA | College/University | Alcohol | 250 | 70% | NR | 21.0 |
| Zamboanga | 2019 | USA | College/University | Alcohol | 2449 | 41% | NR | 18.8 |
| Zill | 2019 | EU | Community | Alcohol | 608 | 56% | NR | 40.4 |

^Note: NR = Not reported; a = Median used^
