# Supplementary material for: Benefits of Digital Health Resources for Substance Use Concerns in Women: Scoping Review
Source: JMIR Ment Health. 2021 Jun 7;8(6):e25952. doi: 10.2196/25952 (PMC8218208; doi:10.2196/25952)
Supplement: Multimedia Appendix 3 [file mental_v8i6e25952_app3.docx]

Multimedia Appendix 3: Study design characteristics.

| Author | Year | Randomization | Primary Outcome | Treatment Duration | Follow-Up Duration | ITT Analyses |
| --- | --- | --- | --- | --- | --- | --- |
| Acosta | 2017 | Yes | PTSD Severity^b^ | 12 weeks | 1, 3 months | Yes |
| Acosta | 2012 | Yes | Retention^b^ | 12 months | NA | Yes |
| Acuff | 2019 | Yes | Alcohol induced blackout | 1 session | 1, 6, & 12 months | No |
| Aharonovich | 2017 | Yes | Feasibility | 30 days | 1 month | Yes |
| Albertella | 2019 | No | Cannabis use^b^ | 4 weeks | 1 month | Yes |
| Baldin | 2018 | Yes | Binge drinking^b^ | 1 session | 6 months | Yes |
| Barrio | 2017 | No | Alcohol use^b^ | 6 weeks | NA | No |
| Berman | 2019 | Yes | Binge drinking^b^ | 14 weeks | 7, 14, & 20 weeks | No |
| Berman | 2020 | Yes | Alcohol use | 6 months | 6 weeks | No |
| Bertholet | 2019 | Yes | Alcohol use | 1 session | 6 months | Yes |
|  |  |  |  |  |  |  |
| Bertholet | 2017 | Yes | Alcohol use^b^ | 3 months | NA | Yes |
|  |  |  |  |  |  |  |
| Blankers | 2011 | Yes | Alcohol use^b^ | 3 months | 3 & 6 months | Yes |
| Blankers | 2013 | Yes | Treatment Response | 3 months | 3 & 6 months | Yes |
| Bo | 2018 | Yes | Alcohol use | 5 weeks | 6 months | Yes |
| Bock | 2016 | Yes | Feasibility^b^ | 6 weeks | 12 weeks | No |
| Boyle | 2018 | Yes | Drinking norms^b^ | 1 session | 10 & 20 days | No |
| Brendryen | 2017 | Yes | Alcohol use | 1 session, 2 months | 2 & 6 months | Yes |
| Brendryen | 2014 | Yes | Alcohol use | 6 months | 2 & 6 months | Yes |
| Brief | 2018 | Yes | Alcohol use | 8 weeks | 3 months | No |
| Brief | 2013 | Yes | Alcohol use^b^ | 8 weeks | 3 months | No |
| Brooks | 2010 | Yes | Drug Use^b^ | 8 weeks | 2 weeks | Yes |
| Budney | 2011 | Yes | Cannabis abstinence | 12 weeks | NA | Yes |
| Budney | 2015 | Yes | Cannabis abstinence | 12 weeks | 3 & 9 months | Yes |
| Campbell | 2015 | Yes | Abstinence^b^ | 12 weeks | 3 & 6 months | No |
| Campbell | 2017 | Yes | Abstinence^b^ | 12 weeks | 3 & 6 months | No |
| Campbell | 2014 | Yes | Abstinence | 12 weeks | 3 & 6 months | No |
| Campbell | 2016 | Yes | Abstinence | 3 months | 3 & 6 months | No |
| Carey | 2017 | Yes | Alcohol use^b^ | 1 session | 1, 3, & 6 months | No |
| Carey | 2011 | Yes | Alcohol use | 2 hours | 12 months | No |
| Carra | 2016 | No | Binge drinking | 1 session | 2 weeks | No |
| Chiauzzi | 2005 | Yes | Alcohol use | 4 weeks | 3 months | Yes |
| Choo | 2016 | Yes | Feasibility^b^ | 1 session | 3 months | No |
| Christensen | 2014 | Yes | Abstinence | 12 weeks | NA | No |
| Chung | 2016 | Yes | Alcohol use^b^ | 12 weeks | 6 months | No |
| Cochrane | 2015 | Yes | Abstinence | 12 weeks | 6 months | No |
| Collins | 2014 | Yes | Alcohol use^b^ | 1 session | 12 months | No |
| Copeland | 2017 | Yes | Cannabis use^b^ | 1 session | 1 month | No |
| Crane | 2018 | Yes | Alcohol use | 28 days | NA | Yes |
| Cunningham | 2017 | Yes | Alcohol use^b^ | Variable | 6, 12, 24 months | No |
| Cunningham | 2012 | Yes | Alcohol use | Variable | 6 months | No |
| Cunningham | 2010 | Yes | Alcohol use | 1 session | 12 months | No |
| Cunningham | 2012 | Yes | Alcohol use | 1 session | 6 weeks | No |
| Cunningham | 2009 | Yes | Alcohol use | 1 session | 3 & 6 months | No |
| Deady | 2016 | Yes | Depression severity^b^ | 4 weeks | 3 & 6 months | Yes |
| Delrahim-Howlett | 2011 | Yes | Alcohol use | 1 session | 1 & 2 months | No |
| DeMartini | 2018 | Yes | Feasibility | 8 weeks | NA | No |
| Doumas | 2009 | Yes | Alcohol use | 1 session | 1 month | Yes |
| Dulin | 2017 | Yes | Cravings^b^ | 6 weeks | NA | No |
| Dulin | 2014 | Yes | Alcohol use | 6 weeks | NA | No |
| Dunn | 2020 | Yes | Alcohol use^b^ | 1 session | 4 weeks | No |
| Elison | 2015a | No | Alcohol use^b^ | 12 weeks | NA | No |
| Elison | 2015b | No | Substance use^b^ | 12 weeks | NA | No |
| Elison | 2017 | No | Engagement | 8 weeks | NA | No |
| Fazzino | 2016 | Yes | Alcohol use^b^ | 1 session | 1 month | No |
| Finfgeld-Connett | 2008 | Yes | Alcohol use | 90 days | NA | No |
| Gajecki | 2017 | Yes | Alcohol use | 12 weeks | NA | No |
| Gajecki | 2014 | Yes | Blood alcohol^b^ | 7 weeks | NA | No |
| Geisner | 2015 | Yes | Alcohol harms^b^ | 1 session | 1 month | Yes |
| Gilmore | 2016 | Yes | Heavy drinking | 1 session | 3 months | No |
| Gilmore | 2018 | Yes | Sexual assault^b^ | 1 session | 3 months | No |
| Gilmore | 2015 | Yes | Sexual assault^b^ | 1 session | 3 months | No |
| Glass | 2017 | Yes | Alcohol use | 8 months | 4 months | No |
| Gonzales | 2014 | Yes | Relapse | 12 weeks | 3 months | No |
| Gonzales-Castaneda | 2019 | Yes | Relapse | 12 weeks | 3 months | Yes |
| Gonzalez | 2015 | No | Alcohol use^b^ | 6 weeks | NA | NA |
| Guarino | 2016 | Yes | Feasibility^b^ | 12 weeks | NA | No |
| Guillemont | 2017 | Yes | Alcohol use | 1 session | 6 weeks | No |
| Gustafson | 2014 | Yes | Risky drinking | 8 months | 4 months | Yes |
| Haskins | 2017 | Yes | Treatment initiation^b^ | 1 session | 1 & 3 months | Yes |
| Haug | 2015 | Yes | Alcohol use^b^ | 6 months | NA | Yes |
| Hansen | 2012 | Yes | Alcohol use | 1 session | 6 & 12 months | Yes |
| Hester | 2011 | Yes | Alcohol use | 3 months | 3, 6, & 12 months | Yes |
| Hester | 2012 | Yes | Alcohol use | Single session | 1 & 12 months | No |
| Hester | 2009 | Yes | Abstinence^b^ | 9 weeks | 3, 6, & 12 months | Yes |
| Hester | 2013 | Yes | Abstinence^b^ | 3 months | 3 months | Yes |
| Hester | 2005 | Yes | Alcohol use | 1 session | 8 weeks & 12 months | No |
| Hunter | 2017 | Yes | Harmful drinking | 1 session | 3 & 12 months | No |
| Ingersoll | 2018 | Yes | Risky drinking^b^ | 9 weeks | 6 months | Yes |
| Jo S-J | 2019 | Yes | Alcohol use | 4 weeks | 4 weeks | Yes |
| Johansson | 2017 | No | Low-risk use^b^ | 10 weeks | NA | No |
| Johnston | 2019 | No | Treatment retention^b^ | 6 months | NA | No |
| Jonas | 2018 | Yes | Cannabis use | 28 days & 50 days | 3, 6, & 12 months | Yes |
| Jonas | 2019 | Yes | Cannabis use | 28 days & 50 days | 3, 6, & 12 months | Yes |
| Kazemi | 2020 | Yes | Alcohol use^b^ | 2 weeks | 6 weeks | No |
| Khadjesari | 2014 | Yes | Alcohol use | 1 session | 3 months | Yes |
| Kiluk | 2016 | Yes | Alcohol use | 8 weeks | 1, 3, & 6 months | Yes |
| Kiluk | 2018 | Yes | Substance use | 12 weeks | 1, 3, & 6 months | Yes |
| Kim | 2016 | Yes | Abstinence | 52 weeks | NA | No |
| Klein | 2013 | No | Engagement | 18 months | NA | No |
| Klein | 2012 | No | Engagement | 18 months | NA | No |
| Kypri | 2009 | Yes | Alcohol use | 1 session | 1 & 6 months | Yes |
| Kypri | 2008 | Yes | Alcohol use | 1 session, 3 sessions | 6 & 12 months | Yes |
| Kypri | 2013 | Yes | Alcohol use | 1 session | 5 months | Yes |
| Kypri | 2004 | Yes | Alcohol use | 1 session | 6 weeks & 6 months | No |
| Leeman | 2016 | Yes | Alcohol use^b^ | 1 session | 1 & 6 months | Yes |
| Levesque | 2017 | Yes | Coping | 12 weeks | 6 months | Yes |
| Lewis | 2019 | Yes | Sexual behaviour^b^ | 1 session | 1 & 6 months | Yes |
| Liang | 2018 | Yes | Drug use | 4 weeks | NA | No |
| Linowski | 2016 | Yes | Alcohol use^b^ | 1 session | 3 & 9 months | No |
| Livingston | 2020 | No | Alcohol use^b^ | 1 session | 1, 3, & 6 months | No |
| Mariano | 2019 | Yes | Social functioning | 12 weeks | 3 & 6 months | No |
| Marsch | 2014 | Yes | Abstinence | 52 weeks | NA | Yes |
| Mason | 2014 | Yes | Alcohol use^b^ | 4 days | 1 month | No |
| Mason | 2020 | Yes | Cannabis use^b^ | 4 weeks | 2 & 3 months | No |
| Miller | 2018 | Yes | Alcohol use^b^ | 1 session | 1 month | No |
| Muench | 2017 | Yes | Alcohol use^b^ | 12 weeks | NA | Yes |
| Murphy | 2010 | Yes | Alcohol use | 1 session | 1 month | No |
| Murphy | 2015 | Yes | Alcohol use^b^ | 1 session | 1 & 6 months | No |
| Murray | 2012 | No | Engagement | Variable | 12 months | No |
| Neighbors | 2010 | Yes | Alcohol use | 1 session, 4 sessions | 6, 12, 18, & 24 months | Yes |
| Osilla | 2015 | Yes | Acceptance | 1 session | 3 months | Yes |
| Paris | 2018 | Yes | Substance use | 8 weeks | 1, 3, & 6 months | Yes |
| Pedersen | 2017 | Yes | Alcohol use^b^ | 1 session | 1 month | No |
| Possemato | 2019 | Yes | Alcohol use^b^ | 12 weeks | 24 Weeks | No |
| Riper | 2008 | Yes | Alcohol use | 6 weeks | 6 months | Yes |
| Rooke | 2014 | Yes | Cannabis use^b^ | 6 weeks | 3 months | No |
| Schaub | 2019 | Yes | Cocaine use | 6 weeks | 6 months | No |
| Schaub | 2012 | Yes | Cocaine dependence | 6 weeks | 4 weeks, 6 weeks & 6 months | No |
| Schulz | 2013 | Yes | Alcohol use^b^ | 3 sessions | 6 months | Yes |
| Sharpe | 2019 | Yes | Alcohol harms | 4 weeks | 3, 6 & 12 months | Yes |
| Sharpe | 2018 | Yes | Alcohol use | 4 weeks | 3, 6 & 12 months | Yes |
| Shrier | 2014 | No | Feasibility^b^ | 2 weeks | 3 months | No |
| Shulman | 2018 | Yes | Abstinence | 12 weeks | NA | No |
| Sinadinovic | 2014a | Yes | Alcohol use | 12 months | NA | Yes |
| Sinadinovic | 2014b | Yes | Drug use | 3 months | 12 months | Yes |
| Sinadinovic | 2020 | Yes | Cannabis use | 12 weeks | NA | Yes |
| Sinadinovic | 2012 | Yes | Substance use | 1 session | 3 & 6 months | Yes |
| Steers | 2016 | Yes | Alcohol use^b^ | 1 session | 2 weeks | No |
| Suffoletto | 2014 | Yes | Binge drinking^b^ | 12 weeks | 3 months | No |
| Suffoletto | 2015 | Yes | Binge drinking^b^ | 12 weeks | 3, 6 & 9 months | No |
| Suffoletto | 2020 | Yes | Cravings^b^ | 14 weeks | NA | No |
| Suffoletto | 2012 | Yes | Engagement | 12 weeks | 3 months | No |
| Sundstrom | 2016 | Yes | Alcohol use | 10 weeks | NA | Yes |
| Sundstrom | 2020 | Yes | Alcohol use | 12 weeks | 6 months | Yes |
| Sundstrom | 2017 | Yes | Alcohol use | 12 weeks | 3 months | Yes |
| Susukida | 2018 | Yes | Abstinence | 12 weeks | 3 & 6 months | No |
| Tahaney | 2017 | Yes | Alcohol use | 1 month | NA | No |
| Tait | 2015 | Yes | Substance use | 3 weeks | 6 months | Yes |
| Tait | 2019 | Yes | Alcohol use | 1 month | 1 & 3 months | Yes |
| Takano | 2020 | Yes | Abstinence | 8 weeks | 2, 5, & 8 months | Yes |
| Teeters | 2018 | Yes | Driving after drinking | 1 session | 3 months | No |
| Tensil | 2013 | Yes | Alcohol use^b^ | 14 days | 6 weeks & 3 months | Yes |
| Tetrault | 2020 | Yes | Feasibility^b^ | 8 weeks | 8 weeks | No |
| Tofighi | 2016 | Yes | Acceptability | 12 weeks | 6 months | No |
| Vaezazizi | 2019 | Yes | Substance use | 12 weeks | NA | No |
| Van Lettow | 2015 | Yes | Alcohol use^b^ | 1 session | 1 & 6 months | No |
| Voogt | 2014 | Yes | Alcohol use^b^ | 1 session | 6 months | Yes |
| Voogt | 2013a | Yes | Alcohol use | 1 session | Weekly for 6 months | Yes |
| Voogt | 2013b | Yes | Alcohol use^b^ | 1 session | 1 & 6 months | Yes |
| Wallace | 2017 | Yes | Harmful drinking | 1 session | 3 & 12 months | Yes |
| Walukevich-Dienst | 2019 | Yes | Cannabis use | 1 session | 1 month | No |
| Walukevich-Dienst | 2020 | Yes | Cannabis use | 1 session | 1 month | No |
| Ward | 2019 | No | Treatment goals | Variable | NR | No |
| Wilks | 2018 | Yes | Suicidal ideation^b^ | 8 weeks | 1, 2, & 4 months | Yes |
| Wilson | 2015 | No | Treatment effectiveness^b^ | 6 months | NA | No |
| Witkiewitz | 2014 | Yes | Alcohol use^b^ | 2 weeks | 1 month | Yes |
| Young | 2019 | Yes | Alcohol use^b^ | 1 session | 1 month | No |
| Zamboanga | 2019 | Yes | Protective strategies^b^ | 3 days | 1 & 4 months | Yes |
| Zill | 2019 | Yes | Alcohol use | 6 months | NA | Yes |

^Note: NR = Not reported; NA = Not applicable; b = Primary outcome not specified, first outcome reported indicated^
