# Supplementary material for: Benefits of Digital Health Resources for Substance Use Concerns in Women: Scoping Review
Source: JMIR Ment Health. 2021 Jun 7;8(6):e25952. doi: 10.2196/25952 (PMC8218208; doi:10.2196/25952)
Supplement: Multimedia Appendix 4 [file mental_v8i6e25952_app4.docx]

Multimedia Appendix 4: Intervention characteristics.

| Author | Year | Intervention Name | Intervention N | Language of Intervention | Platform (Online, Mobile, Combined) | Single vs. Multi-Session | Intervention Effective? | Intervention Effective in Women/  Females? |
| --- | --- | --- | --- | --- | --- | --- | --- | --- |
| Acosta | 2017 | Thinking Forward | 81 | English | Online | Multi | Yes | NR |
| Acosta | 2012 | TES | 80 | English | Online | Multi | Yes | NR |
| Acuff | 2019 | e-CHUG | 45 | English | Online | Single | No | NR |
| Aharonovich | 2017 | HealthCall-S | 23 | English | Mobile | Multi | Yes | NR |
| Albertella | 2019 | APTT App | 111 | English | Mobile | Multi | Yes | NR |
| Baldin | 2018 | Study Specific Intervention | 224 | Spanish | Online | Single | Yes | NR |
| Barrio | 2017 | SIDEAL app | 24 | Spanish | Mobile | Multi | Yes | NR |
| Berman | 2019 | PartyPlanner | 722 | Swedish | Mobile | Multi | Yes | NR |
|  |  | Promillekoll | 722 | Swedish | Mobile | Multi | Yes | NR |
| Berman | 2020 | Telecoach | 42 | English | Mobile | Multi | Yes | NR |
|  |  | Promillekoll | 47 | Swedish | Mobile | Multi | Yes | NR |
| Bertholet | 2017 | Alcooquizz | 130 | Multiple | Combined | Multi | Yes | Yes |
| Bertholet | 2019 | Alcooquizz | 461 | Multiple | Mobile | Single | No | NR |
| Blankers | 2011 | Self-help Alcohol Online (SAO) | 68 | NR | Online | Multi | Yes | NR |
| Blankers | 2013 | Self-help Alcohol Online (SAO) | 68 | NR | Online | Multi | Yes | NR |
| Bo | 2018 | GET.ON Clever weniger trinken | 146 | German | Online | Multi | Yes | Yes |
| Bock | 2016 | TMAP | 31 | English | Mobile | Multi | Yes | NR |
| Boyle | 2018 | Study Specific Intervention | 141 | English | Online | Single | Yes | NR |
| Brendryen | 2017 | Balance | 43 | Norweigen | Online | Multi | No | NR |
| Brendryen | 2014 | Balance | 125 | Norweigen | Online | Multi | Yes | NR |
| Brief | 2018 | VetChange | 404 | English | Online | Multi | Yes | NR |
| Brief | 2013 | VetChange | 600 | English | Online | Multi | Yes | NR |
| Brooks | 2010 | TES | 14 | English | Online | Multi | Yes | NR |
| Budney | 2011 | Study Specific Intervention | 16 | English | Online | Multi | Yes | NR |
| Budney | 2015 | Adapted TES | 30 | English | Online | Multi | Yes | NR |
| Campbell | 2015 | TES | 255 | English | Online | Multi | Yes | Yes |
| Campbell | 2017 | TES | 255 | English | Online | Multi | Yes | NR |
| Campbell | 2014 | TES | 255 | English | Online | Multi | Yes | NR |
| Campbell | 2016 | Overcoming Addictions | 19 | English | Online | Multi | Yes | NR |
| Carey | 2017 | College Drinker's Check Up | 190 | English | Online | Single | Yes | NR |
| Carey | 2011 | Alcohol 101 Edu | 167 | English | Online | Single | Yes | Yes |
| Carra | 2016 | D-ARIANNA | 590 | Multiple | Mobile | Multi | Yes | NR |
| Chiauzzi | 2005 | MSB: Alcohol | 131 | English | Online | Single | Yes | Yes |
| Choo | 2016 | BSAFER | 21 | English | Online | Single | Yes | Yes |
| Christensen | 2014 | CRA+ | 92 | English | Online | Multi | Yes | NR |
| Chung | 2016 | TRAC | 384 | English | Mobile | Multi | Yes | NR |
| Cochrane | 2015 | TES | 255 | English | Online | Multi | Yes | NR |
| Collins | 2014 | Web Decisional balance feedback | 251 | English | Online | Single | Yes | NR |
|  |  | DBF | 242 | English | Online | Single | Yes | NR |
| Copeland | 2017 | Grassessment (Brief) | 156 | English | Online | Single | Yes | NR |
|  |  | Grassessment (Intensive) | 131 | English | Online | Single | Yes | NR |
| Crane | 2018 | Drink Less | 672 | English | Mobile | Multi | Yes | NR |
| Cunningham | 2017 | CheckYourDrinking.net | 239 | English | Online | Single | No | NR |
|  |  | AlcoholHelpCentre.net | 251 | English | Online | Multi | No | NR |
| Cunningham | 2012 | Check Your Drinking | 87 | English | Online | Single | Yes | NR |
|  |  | AlcoholHelpCenter.net | 83 | English | Online | Multi | Yes | NR |
| Cunningham | 2010 | Check Your Drinking | 92 | English | Online | Single | No | NR |
| Cunningham | 2012 | Check Your Drinking University Version | 211 | English | Online | Single | No | NR |
| Cunningham | 2009 | Check Your Drinking | 92 | English | Online | Single | Yes | NR |
| Deady | 2016 | The DEAL Project | 60 | English | Online | Multi | Yes | NR |
| Delrahim-Howlett | 2011 | e-CheckUp | 75 | English | Online | Single | Yes | Yes |
| DeMartini | 2018 | Study Specific Intervention | 8 | English | Mobile | Multi | Yes | NR |
| Doumas | 2009 | Check Your Drinking | 76 | English | Online | Single | Yes | NR |
| Dulin | 2017 | LBMI-A | 28 | English | Mobile | Multi | Yes | NR |
| Dulin | 2014 | LBMI-A | 28 | English | Mobile | Multi | Yes | NR |
| Dunn | 2020 | ECALC | 58 | English | Online | Multi | Yes | Yes |
| Elison | 2015a | BFO | 300 | English | Online | Multi | Yes | NR |
| Elison | 2015b | BFO | 393 | English | Online | Multi | Yes | Yes |
| Elison | 2017 | BFO | 2311 | English | Online | Multi | Yes | NR |
| Fazzino | 2016 | e-CHUG | 856 | English | Online | Single | No | Yes |
| Finfgeld-Connett | 2008 | WebCT | 34 | English | Online | Multi | Yes | Yes |
| Gajecki | 2017 | TeleCoach | 93 | English | Mobile | Multi | Yes | Yes |
| Gajecki | 2014 | PartyPlanner | 643 | Swedish | Mobile | Multi | No | NR |
|  |  | Promillekoll | 639 | Swedish | Mobile | Multi | No | NR |
| Geisner | 2015 | Study specific Intervention (Alcohol only) | 84 | English | Online | Single | No | NR |
|  |  | Study Specific Intervention (Alcohol & Mood) | 85 | English | Online | Single | No | NR |
| Gilmore | 2016 | Study Specific Intervention (Alcohol only) | 53 | English | Online | Single | No | No |
|  |  | Study Specific Intervention (Alcohol & Sexual Assault Risk Reduction) | 52 | English | Online | Single | No | No |
| Gilmore | 2018 | Study Specific Intervention (Alcohol only) | 53 | English | Online | Single | No | No |
|  |  | Study Specific Intervention (Alcohol & Sexual Assault Risk Reduction) | 52 | English | Online | Single | Yes | Yes |
| Gilmore | 2015 | Study Specific Intervention (Alcohol only) | 53 | English | Online | Single | Yes | Yes |
|  |  | Study Specific Intervention (Alcohol & Sexual Assault Risk Reduction) | 52 | English | Online | Single | No | No |
| Glass | 2017 | A-CHESS | 170 | English | Mobile | Multi | Yes | NR |
| Gonzales | 2014 | ESQYIR text messaging | 40 | English | Mobile | Multi | Yes | NR |
| Gonzales-Castaneda | 2019 | ESQYIR text messaging | 40 | English | Mobile | Multi | Yes | NR |
| Gonzalez | 2015 | LBMI-A | 28 | English | Mobile | Multi | Yes | Yes |
|  |  | Drinker's Check Up | 26 | English | Online | Single | Yes | Yes |
| Guarino | 2016 | Check-In Program | 25 | English | Mobile | Multi | Yes | NR |
| Guillemont | 2017 | Alcoometre self-help Intervention | 734 | Other | Online | Multi | Yes | NR |
| Gustafson | 2014 | A-CHESS | 170 | English | Mobile | Multi | Yes | NR |
| Hansen | 2012 | Study Specific Intervention (PNF) | 476 | English | Online | Single | No | No |
|  |  | Study Specific Intervention (BA) | 450 | English | Online | Single | No | No |
| Haskins | 2017 | HERA | 97 | English | Online | Single | No | NR |
| Haug | 2015 | Study Specific Intervention | 25 | English | Mobile | Multi | No | NR |
| Hester | 2011 | ModerateDrinking.com | 40 | English | Online | Multi | Yes | NR |
| Hester | 2012 | College Drinker's Check Up | 65 | English | Online | Single | Yes | NR |
|  |  |  | 42 | English | Online | Single | Yes | NR |
| Hester | 2009 | ModerateDrinking.com | 40 | English | Online | Multi | Yes | NR |
| Hester | 2013 | Overcoming Addictions | 19 | English | Online | Multi | Yes | NR |
| Hester | 2005 | Drinker's Check-up (Immediate) | 35 | English | Online | Single | Yes | NR |
|  |  | Drinker's Check-up (Delayed) | 26 | English | Online | Single | Yes | NR |
| Hunter | 2017 | Down your drink | 347 | English | Online | Single | Yes | NR |
| Ingersoll | 2018 | CARRII intervention | 37 | English | Online | Multi | Yes | Yes |
| Jo S-J | 2019 | on-BEAM | 748 | English | Online | Multi | Yes | NR |
| Johansson | 2017 | e-Change | 4165 | Swedish | Online | Multi | Yes | NR |
| Johnston | 2019 | A-CHESS | 98 | English | Mobile | Multi | Yes | Yes |
| Jonas | 2019 | Quit the Shit | 534 | English | Online | Multi | Yes | NR |
| Jonas | 2018 | Quit the Shit (50 days w/ chat) | 136 | English | Online | Multi | Yes | NR |
|  |  | Quit the Shit (28 days w/ chat) | 135 | English | Online | Multi | Yes | NR |
|  |  | Quit the Shit (50 days w/o chat) | 131 | English | Online | Multi | Yes | NR |
|  |  | Quit the Shit (28 days w/o chat) | 132 | English | Online | Multi | Yes | NR |
| Kazemi | 2020 | Study Specific Intervention | 151 | English | Mobile | Multi | Yes | NR |
| Khadjesari | 2014 | Study Specific Intervention | 659 | English | Online | Single | No | NR |
| Kiluk | 2016 | CBT4CBT | 46 | English | Online | Multi | Yes | NR |
| Kiluk | 2018 | CBT4CBT | 38 | English | Online | Multi | Yes | NR |
| Kim | 2016 | TES | 80 | English | Online | Multi | Yes | NR |
| Klein | 2013 | MORE | 1682 | English | Online | Multi | Yes | NR |
| Klein | 2012 | MORE | 1124 | English | Online | Multi | Yes | NR |
| Kypri | 2009 | Web-based motivational feedback | 1251 | English | Online | Single | Yes | NR |
| Kypri | 2008 | Study Specific Intervention (Single-dose e-SBI) | 138 | English | Online | Single | Yes | NR |
|  |  | Study Specific Intervention (Multi-dose e-SBI) | 145 | English | Online | Multi | Yes | NR |
| Kypri | 2013 | Study Specific Intervention | 939 | English | Online | Single | Yes | NR |
| Kypri | 2004 | Study Specific Intervention | 51 | English | Online | Single | Yes | NR |
| Leeman | 2016 | THRIVE | 135 | English | Online | Single | Yes | NR |
| Levesque | 2017 | TES | 218 | English | Online | Multi | Yes | NR |
| Lewis | 2019 | Study Specific Intervention | 269 | English | Online | Single | Yes | NR |
| Liang | 2018 | S-Health | 50 | English | Mobile | Multi | Yes | NR |
| Linowski | 2016 | BASICS | 175 | English | Online | Single | Yes | Yes |
| Livingston | 2020 | VetChange | 222 | English | Online | Multi | Yes | NR |
| Mariano | 2019 | TES | 255 | English | Online | Multi | No | No |
| Marsch | 2014 | TES | 80 | English | Online | Multi | Yes | NR |
| Mason | 2020 | PNC-Text | 51 | English | Mobile | Multi | Yes | NR |
| Mason | 2014 | Study Specific Intervention | 8 | English | Mobile | Multi | Yes | NR |
| Miller | 2018 | Study Specific Intervention | 285 | English | Online | Single | Yes | NR |
| Muench | 2017 | Study Specific Intervention | 146 | English | Mobile | Multi | Yes | NR |
| Murphy | 2010 | e-CHUG | 45 | English | Online | Single | No | No |
| Murphy | 2015 | e-CHUG | 45 | English | Online | Single | Yes | NR |
| Murray | 2012 | Down Your Drink | 19 | English | Online | Single | Yes | NR |
| Neighbors | 2012 | Study Specific Intervention (Single Gender-Specific Personalized Feedback) | 163 | English | Online | Single | Yes | Yes |
|  |  | Study Specific Intervention (Biannual Gender-Specific PNF) | 164 | English | Online | Multi | Yes | Yes |
|  |  | Study Specific Intervention (Single Gender-Nonspecific PNF) | 164 | English | Online | Single | Yes | Yes |
|  |  | Study Specific Intervention (Biannual Gender-Nonspecific PNF) | 163 | English | Online | Multi | Yes | Yes |
| Osilla | 2015 | Study Specific Intervention | 54 | Multiple | Online | Single | No | NR |
| Paris | 2018 | CBT4CBT | 43 | Spanish | Online | Multi | Yes | NR |
| Pedersen | 2017 | Study Specific Intervention | 393 | English | Online | Single | Yes | NR |
| Possemato | 2019 | Thinking Forward | 30 | English | Online | Multi | Yes | NR |
| Riper | 2008 | Drinking Less | 130 | Dutch | Online | Multi | Yes | NR |
| Rooke | 2014 | Reduce your Use | 230 | English | Online | Multi | Yes | NR |
| Schaub | 2019 | Snow Control 2.0 | 222 | English | Online | Multi | Yes | NR |
| Schaub | 2012 | Snow Control | 96 | German | Online | Multi | No | NR |
| Schulz | 2013 | Alcohol Everything Within Limits (Alternating Feedback) | 132 | German | Online | Multi | Yes | NR |
|  |  | Alcohol Everything Within Limits (Summative Feedback) | 181 | German | Online | Multi | Yes | NR |
| Sharpe | 2019 | ‘YourCall’ text message | 205 | Multiple | Mobile | Multi | No | NR |
| Sharpe | 2018 | ‘YourCall’ text message | 299 | Multiple | Mobile | Multi | Yes | NR |
| Shrier | 2014 | MOMENT | 27 | English | Mobile | Multi | No | NR |
| Shulman | 2018 | TES | 255 | English | Online | Multi | Yes | NR |
| Sinadinovic | 2014a | e.Screen | 211 | Swedish | Online | Single | Yes | NR |
| Sinadinovic | 2014b | eScreen.se | 211 | Swedish | Online | Single | Yes | NR |
|  |  | Alkoholhjalpen.se | 212 | Swedish | Online | Multi | Yes | NR |
| Sinadinovic | 2020 | Cannabishjälpen | 151 | Swedish | Online | Multi | No | NR |
| Sinadinovic | 2012 | eScreen.se | 101 | Swedish | Online | Single | No | NR |
| Steers | 2016 | Study Specific Intervention | 108 | English | Online | Single | Yes | NR |
| Suffoletto | 2014 | Study Specific Intervention | 580 | English | Mobile | Multi | Yes | NR |
| Suffoletto | 2015 | Study Specific Intervention | 580 | English | Mobile | Multi | Yes | NR |
| Suffoletto | 2020 | Study Specific Intervention | 127 | English | Mobile | Multi | Yes | NR |
| Suffoletto | 2012 | Study Specific Intervention | 15 | English | Mobile | Multi | Yes | NR |
| Sundstrom | 2020 | ePlus Program | 72 | English | Online | Multi | Yes | NR |
|  |  | e-Change | 71 | English | Online | Multi | Yes | NR |
| Sundstrom | 2017 | ePlus Program | 13 | English | Online | Multi | Yes | NR |
| Sundstrom | 2016 | Therapy Alcohol Online (self-help group) | 40 | Swedish | Online | Multi | Yes | NR |
|  |  | Therapy Alcohol Online (w/ asynchronous therapist-messaging) | 20 | Swedish | Online | Multi | Yes | NR |
|  |  | Therapy Alcohol Online (w/ synchronous therapist chat) | 20 | Swedish | Online | Multi | Yes | NR |
| Susukida | 2018 | TES | 507 | Swedish | Online | Multi | No | No |
| Tahaney | 2017 | eCHUG | 111 | English | Online | Multi | No | NR |
|  |  | eCHUG + text-message adjunct |  | English | Combined | Multi | Yes | NR |
| Tait | 2015 | Breakingtheice | 81 | English | Online | Multi | Yes | NR |
| Tait | 2019 | Daybreak | 793 | English | Mobile | Multi | Yes | Yes |
| Takano | 2020 | e-SMARPP | 23 | Other | Online | Multi | Yes | NR |
| Teeters | 2018 | Study Specific Intervention | 43 | English | Online | Single | Yes | Yes |
| Tensil | 2013 | Change Your Drinking Version 1 | 300 | German | Online | Multi | Yes | NR |
| Tetrault | 2020 | CBT4CBT | 30 | English | Online | Multi | Yes | NR |
| Tofighi | 2016 | TES | 255 | English | Online | Multi | Yes | NR |
| Vaezazizi | 2019 | TES | 255 | English | Online | Multi | Yes | NR |
| Van Lettow | 2015 | Drinktest | 860 | English | Online | Single | Yes | NR |
|  |  | Drinktest + Prototype | 660 | English | Online | Single | Yes | NR |
|  |  | Drinktest + Cue reminder | 597 | English | Online | Single | Yes | NR |
|  |  | Drinktest + Combined (Prototype & Cue reminder) | 517 | English | Online | Single | Yes | NR |
| Voogt | 2014 | WDYD | 456 | English | Online | Single | Yes | NR |
| Voogt | 2013a | WDYD | 456 | Dutch | Online | Single | Yes | NR |
| Voogt | 2013b | WDYD | 456 | Dutch | Online | Single | No | NR |
| Wallace | 2017 | Down your drink | 347 | English | Online | Multi | Yes | NR |
| Walukevich-Dienst | 2019 | Study Specific Intervention | 102 | English | Online | Single | Yes | Yes |
| Walukevich-Dienst | 2020 | Study Specific Intervention | 102 | English | Online | Single | Yes | NR |
| Ward | 2019 | BFO | 1937 | English | Online | Multi | Yes | NR |
| Wilks | 2018 | iDBT-ST | 31 | English | Online | Multi | Yes | NR |
| Wilson | 2015 | DODA | 95 | Other | Online | Multi | Yes | NR |
| Witkiewitz | 2014 | BASICS-Mobile | 32 | English | Mobile | Multi | No | NR |
| Young | 2019 | PNF + Expressive Writing | 63 | English | Online | Single | Yes | NR |
| Zamboanga | 2019 | myPlaybook | 1305 | English | Online | Multi | No | No |
| Zill | 2019 | Vorvida | 306 | German | Online | Multi | Yes | NR |

^NR = Not Reported; TES = Therapeutic Education System; BFO=Breaking Free Online; CBT4CBT=Computer Based Training for Cognitive Behavioural Therapy; BASICS=Brief Alcohol Screening and Intervention of College Students; A-CHESS= Addiction-Comprehensive Health Enhancement Support System; e-CHUG= e-Check Up to Go; APTT=Assess, Plan, Track, and Tips; LBMI-A= Location Based Monitoring Intervention for Alcohol; on-BEAM=online-based Brief Empowerment Program for Alcohol-use Monitor; PNF = Personalized normal feedback; DODA= Deaf Off Drugs & Alcohol Online Program; e-SMARPP= E-Serigaya; Methamphetamine Relapse Prevention Program; TRAC= Text Message Behavioral Intervention to Reduce Alcohol Consumption in Young Adults; ECALC=Expectancy Challenge Alcohol Literacy Curriculum; ESQYIR=Educating and Supporting Inquisitive Youth in Recovery; HERA=Health Evaluation and Referral Assistant; WDYD=What Do You Drink; MORE= My Ongoing Recovery Experience^
