# Supplementary material for: Benefits of Digital Health Resources for Substance Use Concerns in Women: Scoping Review
Source: JMIR Ment Health. 2021 Jun 7;8(6):e25952. doi: 10.2196/25952 (PMC8218208; doi:10.2196/25952)
Supplement: Multimedia Appendix 5 [file mental_v8i6e25952_app5.docx]

Multimedia Appendix 5: Risk of bias.

| Author | Year | Domain 1. Randomization process | Domain 2. Deviations from intended interventions | Domain 3. Missing outcome data | Domain 4. Measurement of the outcome | Domain 5. Selection of the reported result | Domain 6. Overall Bias |
| --- | --- | --- | --- | --- | --- | --- | --- |
| Acosta | 2017 | Low | Some | Low | Some | Some | Some |
| Acosta | 2012 | Low | Low | Low | Low | Some | Low |
| Acuff | 2019 | Low | High | High | Some | Some | High |
| Aharanovich | 2017 | Low | Low | Low | Low | Some | Low |
| Albertella | 2019 | High | Low | Low | High | Low | High |
| Baldin | 2018 | Low | Low | Low | Low | Some | Low |
| Barrio | 2017 | Low | High | High | Some | Low | High |
| Berman | 2019 | Low | Low | Low | Some | Some | Some |
| Berman | 2020 | Low | Some | Low | Low | Low | Low |
| Bertholet | 2019 | Low | Low | Low | Some | Low | Low |
| Bertholet | 2017 | Low | Some | Low | Some | Some | Some |
| Blankers | 2011 | Low | Some | Low | Low | Low | Low |
| Blankers | 2013 | Low | Some | Low | Low | Low | Low |
| Bo | 2018 | Low | Some | Low | Some | Low | Some |
| Bock | 2016 | Low | Some | High | Low | Some | Some |
| Boyle | 2018 | Some | Some | Low | Some | Some | Some |
| Brendryen | 2017 | Low | Some | High | Low | Low | Some |
| Brendryen | 2014 | Low | Some | High | Low | Some | Some |
| Brief | 2018 | Some | High | Low | Low | Some | Some |
| Brief | 2013 | Low | Some | Low | Low | Some | Some |
| Brooks | 2010 | Low | Low | Low | Low | Some | Low |
| Budney | 2015 | Some | Low | Low | Low | Some | Some |
| Budney | 2011 | High | Low | Some | High | Some | High |
| Campbell | 2015 | Low | High | Some | Low | Some | Some |
| Campbell | 2017 | Low | High | High | Low | Low | High |
| Campbell | 2014 | Low | Some | Low | Low | Some | Some |
| Campbell | 2016 | Low | Some | Low | Some | Low | Some |
| Carey | 2017 | Some | High | High | Low | Some | High |
| Carey | 2011 | Some | Some | Low | Low | Some | Some |
| Carra | 2016 | Low | High | High | Low | Some | High |
| Chiauzzi | 2005 | Low | Low | Low | Low | Some | Low |
| Choo | 2016 | Some | High | Some | Low | Some | Some |
| Christensen | 2017 | Some | High | Low | Low | Some | Some |
| Chung | 2016 | Some | Some | Low | Some | Some | Some |
| Cochrane | 2015 | Some | Some | Low | Low | Some | Some |
| Collins | 2014 | Low | Some | Low | Some | Some | Some |
| Copeland | 2017 | Some | High | High | Low | Some | High |
| Crane | 2018 | Some | Low | High | Low | Low | Some |
| Cunningham | 2017 | Low | Some | Low | Low | Low | Low |
| Cunningham | 2012 | Low | Low | Low | Low | Low | Low |
| Cunningham | 2010 | Low | Low | Low | Low | Low | Low |
| Cunningham | 2012 | Low | Low | Some | Low | Low | Low |
| Cunningham | 2009 | Low | Some | Low | Low | Low | Low |
| Deady | 2016 | Low | Some | Low | Some | Some | Some |
| Delrahim-Howlett | 2011 | Low | Some | Low | Low | Some | Some |
| DeMartini | 2018 | Low | Some | Low | Low | Low | Low |
| Doumas | 2009 | Low | Some | Low | Low | Some | Some |
| Dulin | 2017 | High | High | Some | Some | Some | High |
| Dulin | 2014 | Low | High | Some | Some | Some | Some |
| Dunn | 2020 | Low | Some | Low | Low | Some | Some |
| Elison | 2015b | Low | High | Low | Low | Some | Some |
| Elison | 2015a | Low | High | Low | Some | Some | Some |
| Elison | 2017 | Low | High | Low | Low | Some | Some |
| Fazzino | 2016 | Some | Some | Low | Low | Some | Some |
| Finfgeld-Connett | 2008 | Low | Some | Low | Some | Some | Some |
| Gajecki | 2014 | Low | Some | Some | Low | Low | Some |
| Gajecki | 2017 | Low | Some | Low | Low | Low | Low |
| Geisner | 2015 | Some | Some | Low | Low | Some | Some |
| Gilmore | 2016 | Some | High | Low | Some | Some | Some |
| Gilmore | 2018 | Low | High | High | Low | Some | High |
| Gilmore | 2015 | Low | High | High | Low | Some | High |
| Glass | 2017 | Low | Some | Low | Some | Some | Some |
| Gonzales | 2014 | Low | Some | Some | Some | Some | Some |
| Gonzales-Castaneda | 2019 | Low | High | Low | Low | Some | Some |
| Gonzalez | 2015 | Low | High | High | Low | Some | High |
| Guarino | 2016 | Some | Some | Low | Low | Some | Some |
| Guillemont | 2017 | Low | Some | Low | Low | Some | Some |
| Gustafson | 2014 | Low | Some | Low | Low | Low | Low |
| Hansen | 2012 | Low | Low | Low | Low | Low | Low |
| Haskins | 2017 | Low | Some | Low | Low | Low | Low |
| Haug | 2015 | Low | Low | Low | Low | Low | Low |
| Hester | 2011 | Low | Some | Low | Low | Some | Some |
| Hester | 2012a | Low | Some | Low | Low | Some | Some |
|  | 2012b | Low | Some | Low | Low | Some | Some |
| Hester | 2009 | Low | Low | Low | Low | Some | Low |
| Hester | 2013 | Low | Low | Low | Low | Low | Low |
| Hester | 2005 | Low | Some | High | Low | Some | Some |
| Hunter | 2017 | Some | Low | Low | Low | Low | Low |
| Some Ingersoll | 2018 | Low | Some | Low | High | Some | Some |
| Jo S-J | 2019 | Low | Low | Low | Low | Low | Low |
| Johansson | 2017 | High | Some | High | Low | Low | High |
| Johnston | 2019 | High | Some | Low | Low | Some | Some |
| Jonas | 2018 | Low | Some | Some | Low | Low | Some |
| Jonas | 2019 | Some | Some | High | High | Low | High |
| Kazemi | 2020 | Low | Some | Low | Low | Some | Some |
| Khadjesari | 2014 | Low | Low | High | Low | Low | Some |
| Kiluk | 2016 | Low | Some | Low | High | Low | Some |
| Kiluk | 2018 | Low | Some | High | Low | Some | Some |
| Some Kim | 2016 | Some | Some | Some | Some | Some | Some |
| Klein | 2013 | High | Some | Low | Low | Some | Some |
| Klein | 2012 | High | High | Low | Low | Some | High |
| Kypri | 2009 | Low | Low | Low | Low | Low | Low |
| Kypri | 2008 | Low | Low | Low | Low | Some | Low |
| Kypri | 2013 | Low | Low | Low | Low | Low | Low |
| Kypri | 2004 | Low | Some | Some | Low | Some | Some |
| Leeman | 2016 | Some | Some | Low | Some | Some | Some |
| Levesque | 2017 | Some | Low | Low | Low | Some | Some |
| Lewis | 2019 | Low | Some | Low | Low | Some | Some |
| Liang | 2018 | Low | Some | Low | Low | Some | Some |
| Linowski | 2016 | Some | Some | Low | Some | Some | Some |
| Livingston | 2020 | Low | High | Some | Some | Some | Some |
| Mariano | 2019 | Low | High | Some | Low | Low | Some |
| Marsch | 2014 | Some | Some | Some | Low | Some | Some |
| Mason | 2014 | High | Some | Low | Some | Some | Some |
| Mason | 2020 | Some | High | Low | Low | Some | Some |
| Miller | 2018 | Some | Some | Low | Low | Some | Some |
| Muench | 2017 | Low | Low | Low | Low | Some | Low |
| Murphy | 2015 | Low | High | High | Low | Some | High |
| Murphy | 2010 | Low | Some | Low | Low | Some | Some |
| Murray | 2012 | High | Low | Some | Some | Some | Some |
| Neighbors | 2010 | Low | Some | Some | Low | Some | Some |
| Osilla | 2015 | Some | Low | Low | Low | Low | Low |
| Paris | 2018 | Low | Some | Low | Low | Some | Some |
| Pederson | 2017 | Low | Some | Low | Low | Low | Low |
| Possemato | 2019 | Low | High | Low | Low | Some | Some |
| Riper | 2008 | Some | Low | Low | Low | Low | Low |
| Rooke | 2014 | Some | High | Low | High | High | High |
| Schaub | 2019 | Some | High | Low | Low | Some | Some |
| Schaub | 2012 | Low | Low | Low | Low | Low | Low |
| Schulz | 2013 | Some | Low | High | Some | Low | Some |
| Sharpe | 2018 | Low | Low | Low | Low | Low | Low |
| Sharpe | 2019 | Low | Some | Low | Low | Low | Low |
| Shrier | 2014 | Low | Some | Low | Low | Some | Some |
| Shulman | 2018 | Some | High | Low | Low | Some | Some |
| Sinadinovic | 2014a | Low | Some | Some | Low | Low | Some |
| Sinadinovic | 2020 | Low | Low | Low | Low | Low | Low |
| Sinadinovic | 2012 | Some | Low | High | Low | Some | Some |
| Sinadinovic | 2014b | Low | Low | Low | Low | Low | Low |
| Steers | 2016 | Some | High | High | Low | Some | High |
| Suffoletto | 2020 | Some | Some | Low | Low | Some | Some |
| Suffoletto | 2014 | Low | Some | Low | Low | Low | Some |
| Suffoletto | 2015 | Low | Low | Low | Low | Low | Low |
| Suffoletto | 2012 | Low | Some | High | Low | Some | Some |
| Sundstrom | 2017 | High | Low | Low | Low | Some | Some |
| Sundstrom | 2019 | Low | Low | Low | Low | Low | Low |
| Sundstrom | 2016 | Low | Some | Low | Some | Low | Some |
| Susukida | 2018 | Some | Some | Low | Low | High | Some |
| Tahaney | 2017 | Some | Some | Some | Low | Some | Some |
| Tait | 2019 | Low | Some | Low | Low | Low | Low |
| Tait | 2015 | Low | Low | Low | Low | Low | Low |
| Takano | 2020 | Low | Low | Low | Low | Low | Low |
| Teeters | 2018 | Low | Low | Low | Some | Some | Some |
| Tensil | 2013 | Low | Low | Some | Low | Low | Low |
| Tetrault | 2020 | Low | Some | Low | Low | Low | Low |
| Tofighi | 2016 | Some | Some | Low | Low | Some | Some |
| Vaezazizi | 2019 | Some | Some | High | Low | Some | Some |
| van Lettow | 2015 | Low | High | Low | Low | Low | Some |
| Voogt | 2014 | Low | Low | Low | Low | Low | Low |
| Voogt | 2013 | Low | Low | Some | Low | Low | Low |
| Voogt | 2013 | Low | Low | Low | Low | Some | Low |
| Wallace | 2017 | Low | Low | Low | High | Low | Some |
| Walukevich-Dienst | 2019 | Low | High | Low | Low | Some | Some |
| Walukevich-Dienst | 2020 | Low | Some | Low | Low | Some | Some |
| Ward | 2019 | Low | High | Low | Low | Some | Some |
| Wilks | 2019 | Low | High | Low | Low | Low | Some |
| Wilson | 2015 | High | High | High | High | Some | High |
| Witkiewitz | 2014 | High | Some | Low | High | Some | Some |
| Young | 2019 | Some | High | Low | Low | Low | Some |
| Zamboanga | 2019 | Low | Low | Low | Low | Some | Low |
| Zill | 2019 | Some | Low | Low | Low | Low | Low |

^Note: Some = Some Concerns^
