# Supplementary material for: Benefits of Digital Health Resources for Substance Use Concerns in Women: Scoping Review
Source: JMIR Ment Health. 2021 Jun 7;8(6):e25952. doi: 10.2196/25952 (PMC8218208; doi:10.2196/25952)
Supplement: Multimedia Appendix 6 [file mental_v8i6e25952_app6.docx]

Multimedia Appendix 6: References of articles included in the scoping review.

1. Acosta MC, Marsch LA, Xie H, Guarino H, Aponte-Melendez Y. A Web-Based Behavior Therapy Program Influences the Association Between Cognitive Functioning and Retention and Abstinence in Clients Receiving Methadone Maintenance Treatment. J Dual Diagn. 2012;8(4):283–293. PMID: 23671409
2. Acosta MC, Possemato K, Maisto SA, Marsch LA, Barrie K, Lantinga L, et al. Web-Delivered CBT Reduces Heavy Drinking in OEF-OIF Veterans in Primary Care With Symptomatic Substance Use and PTSD. Behav Ther. 2017;48(2):262–276. PMID: 28270335
3. Acuff SF, Voss AT, Dennhardt AA, Borsari B, Martens MP, Murphy JG. Brief Motivational Interventions Are Associated with Reductions in Alcohol-Induced Blackouts Among Heavy Drinking College Students. Alcohol Clin Exp Res. 2019;43(5):988–996. PMID: 30973651
4. Aharonovich E, Stohl M, Cannizzaro D, Hasin D. HealthCall delivered via smartphone to reduce co-occurring drug and alcohol use in HIV-infected adults: A randomized pilot trial. J Subst Abuse Treat. 2017;83:15–26. PMID: 29129192
5. Albertella L, Gibson L, Rooke S, Norberg MM, Copeland J. A smartphone app intervention for adult cannabis users wanting to quit or reduce their use: a pilot evaluation. J Cannabis Res. 2019;1(1):9. PMID: 33526112
6. Baldin YC, Sanudo A, Sanchez ZM. Effectiveness of a web-based intervention in reducing binge drinking among nightclub patrons. Rev Saude Publica. 2018;52:2. PMID: 29364357
7. Barrio P, Ortega L, López H, Gual A. Self-management and Shared Decision-Making in Alcohol Dependence via a Mobile App: a Pilot Study. Int J Behav Med. 2017;24(5):722–727. PMID: 28236288
8. Berman AH, Andersson C, Gajecki M, Rosendahl I, Sinadinovic K, Blankers M. Smartphone Apps Targeting Hazardous Drinking Patterns among University Students Show Differential Subgroup Effects over 20 Weeks: Results from a Randomized, Controlled Trial. J Clin Med. 2019;8(11). PMID: 31661868
9. Berman AH, Molander O, Tahir M, Törnblom P, Gajecki M, Sinadinovic K, et al. Reducing Risky Alcohol Use via Smartphone App Skills Training Among Adult Internet Help-Seekers: A Randomized Pilot Trial. Front Psychiatry. 2020;11:434. PMID: 32536880
10. Bertholet N, Daeppen J-B, McNeely J, Kushnir V, Cunningham JA. Smartphone application for unhealthy alcohol use: A pilot study. Subst Abus. 2017;38(3):285–291. PMID: 28113039
11. Bertholet N, Godinho A, Cunningham JA. Smartphone application for unhealthy alcohol use: Pilot randomized controlled trial in the general population. Drug Alcohol Depend. 2019;195:101–105. PMID: 30611977
12. Blankers M, Koeter MWJ, Schippers GM. Internet therapy versus internet self-help versus no treatment for problematic alcohol use: A randomized controlled trial. J Consult Clin Psychol. 2011;79(3):330–341. PMID: 21534652
13. Blankers M, Koeter MWJ, Schippers GM. Baseline predictors of treatment outcome in Internet-based alcohol interventions: a recursive partitioning analysis alongside a randomized trial. BMC Public Health. 2013;13:455. PMID: 23651767
14. Bock BC, Barnett NP, Thind H, Rosen R, Walaska K, Traficante R, et al. A text message intervention for alcohol risk reduction among community college students: TMAP. Addict Behav. 2016;63:107–113. PMID: 27450909
15. Boß L, Lehr D, Schaub MP, Paz Castro R, Riper H, Berking M, et al. Efficacy of a web-based intervention with and without guidance for employees with risky drinking: results of a three-arm randomized controlled trial. Addiction. 2018;113(4):635–646. PMID: 29105879
16. Boyle SC, Earle AM, McCabe N, LaBrie JW. Increasing Chance-Based Uncertainty Reduces Heavy Drinkers’ Cognitive Reactance to Web-Based Personalized Normative Feedback. J Stud Alcohol Drugs. 2018;79(4):601–610. PMID: 30079876
17. Brendryen H, Johansen A, Duckert F, Nesvåg S. A Pilot Randomized Controlled Trial of an Internet-Based Alcohol Intervention in a Workplace Setting. Int J Behav Med. 2017;24(5):768–777. PMID: 28755326
18. Brendryen H, Lund IO, Johansen AB, Riksheim M, Nesvåg S, Duckert F. Balance--a pragmatic randomized controlled trial of an online intensive self-help alcohol intervention. Addiction. 2014;109(2):218–226. PMID: 24134709
19. Brief DJ, Rubin A, Keane TM, Enggasser JL, Roy M, Helmuth E, et al. Web intervention for OEF/OIF veterans with problem drinking and PTSD symptoms: a randomized clinical trial. J Consult Clin Psychol. 2013;81(5):890–900. PMID: 23875821
20. Brief DJ, Solhan M, Rybin D, Enggasser JL, Rubin A, Roy M, et al. Web-based alcohol intervention for veterans: PTSD, combat exposure, and alcohol outcomes. Psychol Trauma. 2018;10(2):154–162. PMID: 28569525
21. Brooks AC, Ryder D, Carise D, Kirby KC. Feasibility and effectiveness of computer-based therapy in community treatment. J Subst Abuse Treat. 2010;39(3):227–235. PMID: 20667682
22. Budney AJ, Fearer S, Walker DD, Stanger C, Thostenson J, Grabinski M, et al. An initial trial of a computerized behavioral intervention for cannabis use disorder. Drug Alcohol Depend. 2011;115(1–2):74–79. PMID: 21131143
23. Budney AJ, Stanger C, Tilford JM, Scherer EB, Brown PC, Li Z, et al. Computer-assisted behavioral therapy and contingency management for cannabis use disorder. Psychol Addict Behav. 2015;29(3):501–511. PMID: 25938629
24. Campbell ANC, Montgomery L, Sanchez K, Pavlicova M, Hu M, Newville H, et al. Racial/ethnic subgroup differences in outcomes and acceptability of an Internet-delivered intervention for substance use disorders. J Ethn Subst Abuse. 2017;16(4):460–478. PMID: 28368740
25. Campbell ANC, Nunes EV, Matthews AG, Stitzer M, Miele GM, Polsky D, et al. Internet-delivered treatment for substance abuse: a multisite randomized controlled trial. Am J Psychiatry. 2014;171(6):683–690. PMID: 24700332
26. Campbell ANC, Nunes EV, Pavlicova M, Hatch-Maillette M, Hu M-C, Bailey GL, et al. Gender-based Outcomes and Acceptability of a Computer-assisted Psychosocial Intervention for Substance Use Disorders. J Subst Abuse Treat. 2015;53:9–15. PMID: 25613105
27. Campbell W, Hester RK, Lenberg KL, Delaney HD. Overcoming Addictions, a Web-Based Application, and SMART Recovery, an Online and In-Person Mutual Help Group for Problem Drinkers, Part 2: Six-Month Outcomes of a Randomized Controlled Trial and Qualitative Feedback From Participants. J Med Internet Res. 2016;18(10):e262. PMID: 27701064
28. Carey KB, Balestrieri SG, Miller MB, Merrill JE, DiBello AM, Benz MB. Efficacy of the College Drinkers Check-Up for Student Drinkers Living Off Campus. J Stud Alcohol Drugs. 2017;78(4):571–579. PMID: 28728639
29. Carey KB, Carey MP, Henson JM, Maisto SA, DeMartini KS. Brief alcohol interventions for mandated college students: comparison of face-to-face counseling and computer-delivered interventions. Addiction. 2011;106(3):528–537. PMID: 21059184
30. Carrà G, Crocamo C, Bartoli F, Carretta D, Schivalocchi A, Bebbington PE, et al. Impact of a Mobile E-Health Intervention on Binge Drinking in Young People: The Digital-Alcohol Risk Alertness Notifying Network for Adolescents and Young Adults Project. J Adolesc Health. 2016;58(5):520–526. PMID: 27107907
31. Chiauzzi E, Green TC, Lord S, Thum C, Goldstein M. My student body: a high-risk drinking prevention web site for college students. J Am Coll Health. 2005;53(6):263–274. PMID: 15900990
32. Choo EK, Zlotnick C, Strong DR, Squires DD, Tapé C, Mello MJ. BSAFER: A Web-based intervention for drug use and intimate partner violence demonstrates feasibility and acceptability among women in the emergency department. Subst Abus. 2016;37(3):441–449. PMID: 26714233
33. Christensen DR, Landes RD, Jackson L, Marsch LA, Mancino MJ, Chopra MP, et al. Adding an Internet-delivered treatment to an efficacious treatment package for opioid dependence. J Consult Clin Psychol. 2014;82(6):964–972. PMID: 25090043
34. Cochran G, Stitzer M, Campbell ANC, Hu M-C, Vandrey R, Nunes EV. Web-based treatment for substance use disorders: differential effects by primary substance. Addict Behav. 2015;45:191–194. PMID: 25697725
35. Collins SE, Kirouac M, Lewis MA, Witkiewitz K, Carey KB. Randomized controlled trial of web-based decisional balance feedback and personalized normative feedback for college drinkers. J Stud Alcohol Drugs. 2014;75(6):982–992. PMID: 25343656
36. Copeland J, Rooke S, Rodriquez D, Norberg MM, Gibson L. Comparison of brief versus extended personalised feedback in an online intervention for cannabis users: Short-term findings of a randomised trial. J Subst Abuse Treat. 2017;76:43–48. PMID: 28162849
37. Crane D, Garnett C, Michie S, West R, Brown J. A smartphone app to reduce excessive alcohol consumption: Identifying the effectiveness of intervention components in a factorial randomised control trial. Sci Rep. 2018;8(1):4384.
38. Cunningham JA, Hendershot CS, Murphy M, Neighbors C. Pragmatic randomized controlled trial of providing access to a brief personalized alcohol feedback intervention in university students. Addict Sci Clin Pract. 2012;7:21. PMID: 23185985
39. Cunningham JA, Shorter GW, Murphy M, Kushnir V, Rehm J, Hendershot CS. Randomized Controlled Trial of a Brief Versus Extended Internet Intervention for Problem Drinkers. Int J Behav Med. 2017;24(5):760–767. PMID: 27770293
40. Cunningham JA, Wild TC, Cordingley J, van Mierlo T, Humphreys K. A randomized controlled trial of an internet-based intervention for alcohol abusers. Addiction. 2009;104(12):2023–2032. PMID: 19922569
41. Cunningham JA, Wild TC, Cordingley J, Van Mierlo T, Humphreys K. Twelve-month follow-up results from a randomized controlled trial of a brief personalized feedback intervention for problem drinkers. Alcohol Alcohol. 2010;45(3):258–262. PMID: 20150170
42. Cunningham JA. Comparison of two internet-based interventions for problem drinkers: randomized controlled trial. J Med Internet Res. 2012;14(4):e107. PMID: 22954459
43. Deady M, Mills KL, Teesson M, Kay-Lambkin F. An Online Intervention for Co-Occurring Depression and Problematic Alcohol Use in Young People: Primary Outcomes From a Randomized Controlled Trial. J Med Internet Res. 2016;18(3):e71. PMID: 27009465
44. Delrahim-Howlett K, Chambers CD, Clapp JD, Xu R, Duke K, Moyer RJ, et al. Web-based assessment and brief intervention for alcohol use in women of childbearing potential: a report of the primary findings. Alcohol Clin Exp Res. 2011;35(7):1331–1338. PMID: 21410488
45. DeMartini KS, Schilsky ML, Palmer A, Fehon DC, Zimbrean P, O’Malley SS, et al. Text Messaging to Reduce Alcohol Relapse in Prelisting Liver Transplant Candidates: A Pilot Feasibility Study. Alcohol Clin Exp Res. 2018;42(4):761–769. PMID: 29498753
46. Doumas DM, McKinley LL, Book P. Evaluation of two Web-based alcohol interventions for mandated college students. J Subst Abuse Treat. 2009;36(1):65–74. PMID: 18657941
47. Dulin PL, Gonzalez VM. Smartphone-based, momentary intervention for alcohol cravings amongst individuals with an alcohol use disorder. Psychol Addict Behav. 2017;31(5):601–607. PMID: 28703611
48. Dulin PL, Gonzalez VM, Campbell K. Results of a pilot test of a self-administered smartphone-based treatment system for alcohol use disorders: usability and early outcomes. Subst Abus. 2014;35(2):168–175. PMID: 24821354
49. Dunn ME, Fried-Somerstein A, Flori JN, Hall TV, Dvorak RD. Reducing alcohol use in mandated college students: A comparison of a Brief Motivational Intervention (BMI) and the Expectancy Challenge Alcohol Literacy Curriculum (ECALC). Exp Clin Psychopharmacol. 2020;28(1):87–98. PMID: 31008641
50. Elison S, Davies G, Ward J. An Outcomes Evaluation of Computerized Treatment for Problem Drinking using Breaking Free Online. Alcoholism Treatment Quarterly. 2015;33(2):185–196.
51. Elison S, Davies G, Ward J. Effectiveness of Computer-Assisted Therapy for Substance Dependence Using Breaking Free Online: Subgroup Analyses of a Heterogeneous Sample of Service Users. JMIR Ment Health. 2015;2(2):e13. PMID: 26543918
52. Elison S, Jones A, Ward J, Davies G, Dugdale S. Examining effectiveness of tailorable computer-assisted therapy programmes for substance misuse: Programme usage and clinical outcomes data from Breaking Free Online. Addict Behav. 2017;74:140–147. PMID: 28645092
53. Fazzino TL, Rose GL, Helzer JE. An experimental test of assessment reactivity within a web-based brief alcohol intervention study for college students. Addict Behav. 2016;52:66–74. PMID: 26363306
54. Finfgeld-Connett D, Madsen R. Web-based treatment of alcohol problems among rural women. J Psychosoc Nurs Ment Health Serv. 2008;46(9):46–53. PMID: 18823000
55. Gajecki M, Andersson C, Rosendahl I, Sinadinovic K, Fredriksson M, Berman AH. Skills Training via Smartphone App for University Students with Excessive Alcohol Consumption: a Randomized Controlled Trial. Int J Behav Med. 2017;24(5):778–788. PMID: 28224445
56. Gajecki M, Berman AH, Sinadinovic K, Rosendahl I, Andersson C. Mobile phone brief intervention applications for risky alcohol use among university students: a randomized controlled study. Addict Sci Clin Pract. 2014;9:11. PMID: 24985342
57. Geisner IM, Varvil-Weld L, Mittmann AJ, Mallett K, Turrisi R. Brief web-based intervention for college students with comorbid risky alcohol use and depressed mood: does it work and for whom? Addict Behav. 2015;42:36–43. PMID: 25462652
58. Gilmore AK, Bountress KE. Reducing drinking to cope among heavy episodic drinking college women: Secondary outcomes of a web-based combined alcohol use and sexual assault risk reduction intervention. Addict Behav. 2016;61:104–111. PMID: 27262965
59. Gilmore AK, Bountress KE, Selmanoff M, George WH. Reducing Heavy Episodic Drinking, Incapacitation, and Alcohol-Induced Blackouts: Secondary Outcomes of a Web-Based Combined Alcohol Use and Sexual Assault Risk Reduction Intervention. Violence Against Women. 2018;24(11):1299–1313. PMID: 30078370
60. Gilmore AK, Lewis MA, George WH. A randomized controlled trial targeting alcohol use and sexual assault risk among college women at high risk for victimization. Behav Res Ther. 2015;74:38–49. PMID: 26408290
61. Glass JE, McKay JR, Gustafson DH, Kornfield R, Rathouz PJ, McTavish FM, et al. Treatment seeking as a mechanism of change in a randomized controlled trial of a mobile health intervention to support recovery from alcohol use disorders. J Subst Abuse Treat. 2017;77:57–66. PMID: 28476273
62. Gonzales R, Ang A, Murphy DA, Glik DC, Anglin MD. Substance use recovery outcomes among a cohort of youth participating in a mobile-based texting aftercare pilot program. J Subst Abuse Treat. 2014;47(1):20–26. PMID: 24629885
63. Gonzales-Castaneda R, McKay JR, Steinberg J, Winters KC, Yu CHA, Valdovinos IC, et al. Testing mediational processes of substance use relapse among youth who participated in a mobile texting aftercare project. Subst Abus. 2019;1–12. PMID: 31638878
64. Gonzalez VM, Dulin PL. Comparison of a smartphone app for alcohol use disorders with an Internet-based intervention plus bibliotherapy: A pilot study. J Consult Clin Psychol. 2015;83(2):335–345. PMID: 25622202
65. Guarino H, Acosta M, Marsch LA, Xie H, Aponte-Melendez Y. A mixed-methods evaluation of the feasibility, acceptability, and preliminary efficacy of a mobile intervention for methadone maintenance clients. Psychol Addict Behav. 2016;30(1):1–11. PMID: 26618796
66. Guillemont J, Cogordan C, Nalpas B, Nguyen-Thanh V, Richard J-B, Arwidson P. Effectiveness of a web-based intervention to reduce alcohol consumption among French hazardous drinkers: a randomized controlled trial. Health Educ Res. 2017;32(4):332–342. PMID: 28854571
67. Gustafson DH, McTavish FM, Chih M-Y, Atwood AK, Johnson RA, Boyle MG, et al. A smartphone application to support recovery from alcoholism: a randomized clinical trial. JAMA Psychiatry. 2014;71(5):566–572. PMID: 24671165
68. Hansen ABG, Becker U, Nielsen AS, Grønbæk M, Tolstrup JS, Thygesen LC. Internet-based brief personalized feedback intervention in a non-treatment-seeking population of adult heavy drinkers: a randomized controlled trial. J Med Internet Res. 2012;14(4):e98. PMID: 22846542
69. Haskins BL, Davis-Martin R, Abar B, Baumann BM, Harralson T, Boudreaux ED. Health Evaluation and Referral Assistant: A Randomized Controlled Trial of a Web-Based Screening, Brief Intervention, and Referral to Treatment System to Reduce Risky Alcohol Use Among Emergency Department Patients. J Med Internet Res. 2017;19(5):e119. PMID: 28461283
70. Haug S, Lucht MJ, John U, Meyer C, Schaub MP. A pilot study on the feasibility and acceptability of a text message-based aftercare treatment programme among alcohol outpatients. Alcohol Alcohol. 2015;50(2):188–194. PMID: 25600249
71. Hester RK, Delaney HD, Campbell W, Handmaker N. A web application for moderation training: initial results of a randomized clinical trial. J Subst Abuse Treat. 2009;37(3):266–276. PMID: 19339137
72. Hester RK, Delaney HD, Campbell W. ModerateDrinking.Com and moderation management: outcomes of a randomized clinical trial with non-dependent problem drinkers. J Consult Clin Psychol. 2011;79(2):215–224. PMID: 21319896
73. Hester RK, Delaney HD, Campbell W. The college drinker’s check-up: outcomes of two randomized clinical trials of a computer-delivered intervention. Psychol Addict Behav. 2012;26(1):1–12. PMID: 21823769
74. Hester RK, Lenberg KL, Campbell W, Delaney HD. Overcoming Addictions, a Web-based application, and SMART Recovery, an online and in-person mutual help group for problem drinkers, part 1: three-month outcomes of a randomized controlled trial. J Med Internet Res. 2013;15(7):e134. PMID: 23846588
75. Hester RK, Squires DD, Delaney HD. The Drinker’s Check-up: 12-month outcomes of a controlled clinical trial of a stand-alone software program for problem drinkers. J Subst Abuse Treat. 2005;28(2):159–169. PMID: 15780546
76. Hunter R, Wallace P, Struzzo P, Vedova RD, Scafuri F, Tersar C, et al. Randomised controlled non-inferiority trial of primary care-based facilitated access to an alcohol reduction website: cost-effectiveness analysis. BMJ Open. 2017;7(11):e014577. PMID: 29102983
77. Ingersoll K, Frederick C, MacDonnell K, Ritterband L, Lord H, Jones B, et al. A Pilot RCT of an Internet Intervention to Reduce the Risk of Alcohol-Exposed Pregnancy. Alcohol Clin Exp Res. 2018;42(6):1132–1144. PMID: 29741798
78. Jo S-J, Lee HK, Kang K, Joe KH, Lee S-B. Efficacy of a Web-Based Screening and Brief Intervention to Prevent Problematic Alcohol Use in Korea: Results of a Randomized Controlled Trial. Alcohol Clin Exp Res. 2019;43(10):2196–2202. PMID: 31386203
79. Johansson M, Sinadinovic K, Hammarberg A, Sundström C, Hermansson U, Andreasson S, et al. Web-Based Self-Help for Problematic Alcohol Use: a Large Naturalistic Study. Int J Behav Med. 2017;24(5):749–759. PMID: 27900733
80. Johnston DC, Mathews WD, Maus A, Gustafson DH. Using Smartphones to Improve Treatment Retention Among Impoverished Substance-Using Appalachian Women: A Naturalistic Study. Subst Abuse. 2019;13:1178221819861377. PMID: 31312084
81. Jonas B, Tensil M-D, Leuschner F, Strüber E, Tossmann P. Predictors of treatment response in a web-based intervention for cannabis users. Internet Interv. 2019;18:100261. PMID: 31890614
82. Jonas B, Tensil M-D, Tossmann P, Strüber E. Effects of Treatment Length and Chat-Based Counseling in a Web-Based Intervention for Cannabis Users: Randomized Factorial Trial. J Med Internet Res. 2018;20(5):e166. PMID: 29739738
83. Kazemi DM, Borsari B, Levine MJ, Li S, Shehab M, Fang F, et al. Effectiveness of a Theory-Based mHealth Intervention for High-Risk Drinking in College Students. Subst Use Misuse. 2020;55(10):1667–1676. PMID: 32394772
84. Khadjesari Z, Freemantle N, Linke S, Hunter R, Murray E. Health on the web: randomised controlled trial of online screening and brief alcohol intervention delivered in a workplace setting. PLoS One. 2014;9(11):e112553. PMID: 25409454
85. Kiluk BD, Devore KA, Buck MB, Nich C, Frankforter TL, LaPaglia DM, et al. Randomized Trial of Computerized Cognitive Behavioral Therapy for Alcohol Use Disorders: Efficacy as a Virtual Stand-Alone and Treatment Add-On Compared with Standard Outpatient Treatment. Alcohol Clin Exp Res. 2016;40(9):1991–2000. PMID: 27488212
86. Kiluk BD, Nich C, Buck MB, Devore KA, Frankforter TL, LaPaglia DM, et al. Randomized Clinical Trial of Computerized and Clinician-Delivered CBT in Comparison With Standard Outpatient Treatment for Substance Use Disorders: Primary Within-Treatment and Follow-Up Outcomes. Am J Psychiatry. 2018;175(9):853–863. PMID: 29792052
87. Kim SJ, Marsch LA, Acosta MC, Guarino H, Aponte-Melendez Y. Can persons with a history of multiple addiction treatment episodes benefit from technology delivered behavior therapy? A moderating role of treatment history at baseline. Addict Behav. 2016;54:18–23. PMID: 26657820
88. Klein AA, Anker JJ. Computer-based recovery support for patients receiving residential treatment for alcohol/drug dependence: relationship between program use and outcomes. Telemed J E Health. 2013;19(2):104–109. PMID: 23215735
89. Klein AA, Slaymaker VJ, Dugosh KL, McKay JR. Computerized continuing care support for alcohol and drug dependence: a preliminary analysis of usage and outcomes. J Subst Abuse Treat. 2012;42(1):25–34. PMID: 21862275
90. Kypri K, Hallett J, Howat P, McManus A, Maycock B, Bowe S, et al. Randomized controlled trial of proactive web-based alcohol screening and brief intervention for university students. Arch Intern Med. 2009;169(16):1508–1514. PMID: 19752409
91. Kypri K, Langley JD, Saunders JB, Cashell-Smith ML, Herbison P. Randomized controlled trial of web-based alcohol screening and brief intervention in primary care. Arch Intern Med. 2008;168(5):530–536. PMID: 18332300
92. Kypri K, McCambridge J, Vater T, Bowe SJ, Saunders JB, Cunningham JA, et al. Web-based alcohol intervention for Māori university students: double-blind, multi-site randomized controlled trial. Addiction. 2013;108(2):331–338. PMID: 22925046
93. Kypri K, Saunders JB, Williams SM, McGee RO, Langley JD, Cashell-Smith ML, et al. Web-based screening and brief intervention for hazardous drinking: a double-blind randomized controlled trial. Addiction. 2004;99(11):1410–1417. PMID: 15500594
94. Leeman RF, DeMartini KS, Gueorguieva R, Nogueira C, Corbin WR, Neighbors C, et al. Randomized controlled trial of a very brief, multicomponent web-based alcohol intervention for undergraduates with a focus on protective behavioral strategies. J Consult Clin Psychol. 2016;84(11):1008–1015. PMID: 27599223
95. Lévesque A, Campbell ANC, Pavlicova M, Hu M-C, Walker R, McClure EA, et al. Coping strategies as a mediator of internet-delivered psychosocial treatment: Secondary analysis from a NIDA CTN multisite effectiveness trial. Addict Behav. 2017;65:74–80. PMID: 27776269
96. Lewis MA, Rhew IC, Fairlie AM, Swanson A, Anderson J, Kaysen D. Evaluating Personalized Feedback Intervention Framing with a Randomized Controlled Trial to Reduce Young Adult Alcohol-Related Sexual Risk Taking. Prev Sci. 2019;20(3):310–320. PMID: 29511966
97. Liang D, Han H, Du J, Zhao M, Hser Y-I. A pilot study of a smartphone application supporting recovery from drug addiction. J Subst Abuse Treat. 2018;88:51–58. PMID: 29606226
98. Linowski SA, DiFulvio GT, Fedorchak D, Puleo E. Effectiveness of an Electronic Booster Session Delivered to Mandated Students. Int Q Community Health Educ. 2016;36(2):123–129. PMID: 26857563
99. Livingston NA, Mahoney CT, Ameral V, Brief D, Rubin A, Enggasser J, et al. Changes in alcohol use, PTSD hyperarousal symptoms, and intervention dropout following veterans’ use of VetChange. Addict Behav. 2020;107:106401. PMID: 32272356
100. Marino LA, Campbell ANC, Pavlicova M, Hu M, Nunes EV. Social functioning outcomes among individuals with substance use disorders receiving internet-delivered community reinforcement approach. Subst Use Misuse. 2019;54(7):1067–1074. PMID: 30849925
101. Marsch LA, Guarino H, Acosta M, Aponte-Melendez Y, Cleland C, Grabinski M, et al. Web-based behavioral treatment for substance use disorders as a partial replacement of standard methadone maintenance treatment. J Subst Abuse Treat. 2014;46(1):43–51. PMID: 24060350
102. Mason M, Benotsch EG, Way T, Kim H, Snipes D. Text messaging to increase readiness to change alcohol use in college students. J Prim Prev. 2014;35(1):47–52. PMID: 24114551
103. Mason MJ. Depressive symptoms moderate cannabis use for young adults in a Text-Delivered randomized clinical trial for cannabis use disorder. Addict Behav. 2020;104:106259. PMID: 31923797
104. Miller MB, DiBello AM, Carey KB, Pedersen ER. Blackouts as a Moderator of Young Adult Veteran Response to Personalized Normative Feedback for Heavy Drinking. Alcohol Clin Exp Res. 2018;42(6):1145–1153. PMID: 29602274
105. Muench F, van Stolk-Cooke K, Kuerbis A, Stadler G, Baumel A, Shao S, et al. A Randomized Controlled Pilot Trial of Different Mobile Messaging Interventions for Problem Drinking Compared to Weekly Drink Tracking. Hills RK, editor. PLoS ONE. 2017;12(2):e0167900.
106. Murphy JG, Dennhardt AA, Skidmore JR, Martens MP, McDevitt-Murphy ME. Computerized versus motivational interviewing alcohol interventions: impact on discrepancy, motivation, and drinking. Psychol Addict Behav. 2010;24(4):628–639. PMID: 21198224
107. Murphy JG, Dennhardt AA, Yurasek AM, Skidmore JR, Martens MP, MacKillop J, et al. Behavioral economic predictors of brief alcohol intervention outcomes. J Consult Clin Psychol. 2015;83(6):1033–1043. PMID: 26167945
108. Murray E, Linke S, Harwood E, Conroy S, Stevenson F, Godfrey C. Widening access to treatment for alcohol misuse: description and formative evaluation of an innovative web-based service in one primary care trust. Alcohol Alcohol. 2012;47(6):697–701. PMID: 22917754
109. Neighbors C, Lewis MA, Atkins DC, Jensen MM, Walter T, Fossos N, et al. Efficacy of web-based personalized normative feedback: a two-year randomized controlled trial. J Consult Clin Psychol. 2010;78(6):898–911. PMID: 20873892
110. Osilla KC, Paddock SM, Leininger TJ, D’Amico EJ, Ewing BA, Watkins KE. A pilot study comparing in-person and web-based motivational interviewing among adults with a first-time DUI offense. Addict Sci Clin Pract. 2015;10:18. PMID: 26334629
111. Palfai TP, Zisserson R, Saitz R. Using personalized feedback to reduce alcohol use among hazardous drinking college students: the moderating effect of alcohol-related negative consequences. Addict Behav. 2011;36(5):539–542. PMID: 21295919
112. Paris M, Silva M, Añez-Nava L, Jaramillo Y, Kiluk BD, Gordon MA, et al. Culturally Adapted, Web-Based Cognitive Behavioral Therapy for Spanish-Speaking Individuals With Substance Use Disorders: A Randomized Clinical Trial. Am J Public Health. 2018;108(11):1535–1542. PMID: 30252519
113. Pedersen ER, Parast L, Marshall GN, Schell TL, Neighbors C. A randomized controlled trial of a web-based, personalized normative feedback alcohol intervention for young-adult veterans. J Consult Clin Psychol. 2017;85(5):459–470. PMID: 28287799
114. Possemato K, Johnson EM, Emery JB, Wade M, Acosta MC, Marsch LA, et al. A pilot study comparing peer supported web-based CBT to self-managed web CBT for primary care veterans with PTSD and hazardous alcohol use. Psychiatr Rehabil J. 2019;42(3):305–313. PMID: 30489140
115. Riper H, Kramer J, Smit F, Conijn B, Schippers G, Cuijpers P. Web-based self-help for problem drinkers: a pragmatic randomized trial. Addiction. 2008;103(2):218–227. PMID: 18199300
116. Rooke SE, Gates PJ, Norberg MM, Copeland J. Applying technology to the treatment of cannabis use disorder: comparing telephone versus Internet delivery using data from two completed trials. J Subst Abuse Treat. 2014;46(1):78–84. PMID: 24051076
117. Schaub MP, Castro RP, Wenger A, Baumgartner C, Stark L, Ebert DD, et al. Web-based self-help with and without chat counseling to reduce cocaine use in cocaine misusers: Results of a three-arm randomized controlled trial. Internet Interv. 2019;17:100251. PMID: 31193584
118. Schaub M, Sullivan R, Haug S, Stark L. Web-based cognitive behavioral self-help intervention to reduce cocaine consumption in problematic cocaine users: randomized controlled trial. J Med Internet Res. 2012;14(6):e166. PMID: 23192752
119. Schulz DN, Candel MJ, Kremers SP, Reinwand DA, Jander A, de Vries H. Effects of a Web-based tailored intervention to reduce alcohol consumption in adults: randomized controlled trial. J Med Internet Res. 2013;15(9):e206. PMID: 24045005
120. Sharpe S, Kool B, Whittaker R, Lee AC, Reid P, Civil I, et al. Effect of a text message intervention to reduce hazardous drinking among injured patients discharged from a trauma ward: a randomized controlled trial. NPJ Digit Med. 2018;1:13. PMID: 31304298
121. Sharpe S, Kool B, Whittaker R, Lee AC, Reid P, Civil I, et al. Effect of a text message intervention on alcohol-related harms and behaviours: secondary outcomes of a randomised controlled trial. BMC Res Notes. 2019;12(1):267. PMID: 31088559
122. Shrier LA, Rhoads A, Burke P, Walls C, Blood EA. Real-time, contextual intervention using mobile technology to reduce marijuana use among youth: a pilot study. Addict Behav. 2014;39(1):173–180. PMID: 24139665
123. Shulman M, Campbell A, Pavlicova M, Hu M-C, Aharonovich E, Nunes EV. Cognitive functioning and treatment outcomes in a randomized controlled trial of internet-delivered drug and alcohol treatment. Am J Addict. 2018;27(6):509–515. PMID: 30091814
124. Sinadinovic K, Johansson M, Johansson A-S, Lundqvist T, Lindner P, Hermansson U. Guided web-based treatment program for reducing cannabis use: a randomized controlled trial. Addict Sci Clin Pract. 2020;15(1):9. PMID: 32070417
125. Sinadinovic K, Wennberg P, Berman AH. Targeting problematic users of illicit drugs with Internet-based screening and brief intervention: a randomized controlled trial. Drug Alcohol Depend. 2012;126(1–2):42–50. PMID: 22613182
126. Sinadinovic K, Wennberg P, Berman AH. Internet-based screening and brief intervention for illicit drug users: a randomized controlled trial with 12-month follow-up. J Stud Alcohol Drugs. 2014;75(2):313–318. PMID: 24650825
127. Sinadinovic K, Wennberg P, Johansson M, Berman AH. Targeting individuals with problematic alcohol use via Web-based cognitive-behavioral self-help modules, personalized screening feedback or assessment only: a randomized controlled trial. Eur Addict Res. 2014;20(6):305–318. PMID: 25300885
128. Steers M-LN, Coffman AD, Wickham RE, Bryan JL, Caraway L, Neighbors C. Evaluation of Alcohol-Related Personalized Normative Feedback With and Without an Injunctive Message. J Stud Alcohol Drugs. 2016;77(2):337–342. PMID: 26997192
129. Suffoletto B, Callaway C, Kristan J, Kraemer K, Clark DB. Text-message-based drinking assessments and brief interventions for young adults discharged from the emergency department. Alcohol Clin Exp Res. 2012;36(3):552–560. PMID: 22168137
130. Suffoletto B, Chung T. Patterns of Change in Weekend Drinking Cognitions Among Non-Treatment-Seeking Young Adults During Exposure to a 12-Week Text Message Intervention. J Stud Alcohol Drugs. 2016;77(6):914–923. PMID: 27797693
131. Suffoletto B, Huber J, Kirisci L, Clark D, Chung T. The effect of SMS behavior change techniques on event-level desire to get drunk in young adults. Psychol Addict Behav. 2020;34(2):320–326. PMID: 31750698
132. Suffoletto B, Kristan J, Callaway C, Kim KH, Chung T, Monti PM, et al. A text message alcohol intervention for young adult emergency department patients: a randomized clinical trial. Ann Emerg Med. 2014;64(6):664-672.e4. PMID: 25017822
133. Suffoletto B, Kristan J, Chung T, Jeong K, Fabio A, Monti P, et al. An Interactive Text Message Intervention to Reduce Binge Drinking in Young Adults: A Randomized Controlled Trial with 9-Month Outcomes. PLoS One. 2015;10(11):e0142877. PMID: 26580802
134. Sundström C, Eék N, Kraepelien M, Fahlke C, Gajecki M, Jakobson M, et al. High- versus low-intensity internet interventions for alcohol use disorders: results of a three-armed randomized controlled superiority trial. Addiction. 2020;115(5):863–874. PMID: 31691413
135. Sundström C, Gajecki M, Johansson M, Blankers M, Sinadinovic K, Stenlund-Gens E, et al. Guided and Unguided Internet-Based Treatment for Problematic Alcohol Use - A Randomized Controlled Pilot Trial. PLoS One. 2016;11(7):e0157817. PMID: 27383389
136. Sundström C, Kraepelien M, Eék N, Fahlke C, Kaldo V, Berman AH. High-intensity therapist-guided internet-based cognitive behavior therapy for alcohol use disorder: a pilot study. BMC Psychiatry. 2017;17(1):197. PMID: 28549424
137. Susukida R, Crum RM, Stuart EA, Mojtabai R. Generalizability of the findings from a randomized controlled trial of a web-based substance use disorder intervention. Am J Addict. 2018;27(3):231–237. PMID: 29569403
138. Tahaney KD, Palfai TP. Text messaging as an adjunct to a web-based intervention for college student alcohol use: A preliminary study. Addict Behav. 2017;73:63–66. PMID: 28478315
139. Tait RJ, McKetin R, Kay-Lambkin F, Carron-Arthur B, Bennett A, Bennett K, et al. Six-month outcomes of a Web-based intervention for users of amphetamine-type stimulants: randomized controlled trial. J Med Internet Res. 2015;17(4):e105. PMID: 25925801
140. Tait RJ, Paz Castro R, Kirkman JJL, Moore JC, Schaub MP. A Digital Intervention Addressing Alcohol Use Problems (the “Daybreak” Program): Quasi-Experimental Randomized Controlled Trial. J Med Internet Res. 2019;21(9):e14967. PMID: 31486406
141. Takano A, Miyamoto Y, Shinozaki T, Matsumoto T, Kawakami N. Effect of a web-based relapse prevention program on abstinence among Japanese drug users: A pilot randomized controlled trial. J Subst Abuse Treat. 2020;111:37–46. PMID: 32087837
142. Teeters JB, Soltis KE, Murphy JG. A Mobile Phone-Based Brief Intervention With Personalized Feedback and Text Messaging Is Associated With Reductions in Driving After Drinking Among College Drinkers. J Stud Alcohol Drugs. 2018;79(5):710–719. PMID: 30422784
143. Tensil M-D, Jonas B, Strüber E. Two fully automated web-based interventions for risky alcohol use: randomized controlled trial. J Med Internet Res. 2013;15(6):e110. PMID: 23742808
144. Tetrault JM, Holt SR, Cavallo DA, O’Connor PG, Gordon MA, Corvino JK, et al. Computerized Cognitive Behavioral Therapy for Substance Use Disorders in a Specialized Primary Care Practice: A Randomized Feasibility Trial to Address the RT Component of SBIRT. J Addict Med. 2020;14(6):e303–e309. PMID: 32371660
145. Tofighi B, Campbell ANC, Pavlicova M, Hu MC, Lee JD, Nunes EV. Recent Internet Use and Associations with Clinical Outcomes among Patients Entering Addiction Treatment Involved in a Web-Delivered Psychosocial Intervention Study. J Urban Health. 2016;93(5):871–883. PMID: 27653383
146. Vaezazizi LM, Campbell ANC, Pavlicova M, Hu M-C, Nunes EV. Understanding site variability in a multisite clinical trial of a technology-delivered psychosocial intervention for substance use disorders. J Subst Abuse Treat. 2019;105:64–70. PMID: 31443894
147. van Lettow B, de Vries H, Burdorf A, Boon B, van Empelen P. Drinker prototype alteration and cue reminders as strategies in a tailored web-based intervention reducing adults’ alcohol consumption: randomized controlled trial. J Med Internet Res. 2015;17(2):e35. PMID: 25653199
148. Voogt CV, Kuntsche E, Kleinjan M, Poelen EAP, Lemmers LACJ, Engels RCME. Using ecological momentary assessment in testing the effectiveness of an alcohol intervention: a two-arm parallel group randomized controlled trial. PLoS One. 2013;8(11):e78436. PMID: 24223806
149. Voogt CV, Kuntsche E, Kleinjan M, Engels RCME. The effect of the “What Do You Drink” web-based brief alcohol intervention on self-efficacy to better understand changes in alcohol use over time: randomized controlled trial using ecological momentary assessment. Drug Alcohol Depend. 2014;138:89–97. PMID: 24613632
150. Voogt CV, Poelen EAP, Kleinjan M, Lemmers LACJ, Engels RCME. The effectiveness of the “what do you drink” web-based brief alcohol intervention in reducing heavy drinking among students: a two-arm parallel group randomized controlled trial. Alcohol Alcohol. 2013;48(3):312–321. PMID: 23303466
151. Wallace P, Struzzo P, Della Vedova R, Scafuri F, Tersar C, Lygidakis C, et al. Randomised controlled non-inferiority trial of primary care-based facilitated access to an alcohol reduction website. BMJ Open. 2017;7(11):e014576. PMID: 29102982
152. Walukevich-Dienst K, Lewis EM, Neighbors C, Green JC, Buckner JD. Online personalized feedback intervention reduces cannabis-related problems among college students with high problem distress. Exp Clin Psychopharmacol. 2021;29(1):14–22. PMID: 32134283
153. Walukevich-Dienst K, Neighbors C, Buckner JD. Online personalized feedback intervention for cannabis-using college students reduces cannabis-related problems among women. Addict Behav. 2019;98:106040. PMID: 31302314
154. Ward J, Elison-Davies S, Davies G, Dugdale S, Jones A. Clinical and demographic patient characteristics, alcohol treatment goal preference and goal attainment during computer-assisted therapy with Breaking Free Online. Journal of Substance Use. 2019;24(6):681–687.
155. Wilks CR, Lungu A, Ang SY, Matsumiya B, Yin Q, Linehan MM. A randomized controlled trial of an Internet delivered dialectical behavior therapy skills training for suicidal and heavy episodic drinkers. J Affect Disord. 2018;232:219–228. PMID: 29499504
156. Witkiewitz K, Desai SA, Bowen S, Leigh BC, Kirouac M, Larimer ME. Development and evaluation of a mobile intervention for heavy drinking and smoking among college students. Psychol Addict Behav. 2014;28(3):639–650. PMID: 25000269
157. Young CM, Neighbors C. Incorporating Writing into a Personalized Normative Feedback Intervention to Reduce Problem Drinking Among College Students. Alcohol Clin Exp Res. 2019;43(5):916–926. PMID: 30817010
158. Zamboanga BL, Merrill JE, Olthuis JV, Milroy JJ, Sokolovsky AW, Wyrick DL. Secondary effects of myPlaybook on college athletes’ avoidance of drinking games or pregaming as a protective behavior strategy: A multisite randomized controlled study. Soc Sci Med. 2019;228:135–141. PMID: 30909157
159. Zill JM, Christalle E, Meyer B, Härter M, Dirmaier J. The Effectiveness of an Internet Intervention Aimed at Reducing Alcohol Consumption in Adults. Dtsch Arztebl Int. 2019;116(8):127–133. PMID: 30940341
